# Fluorinated Zirconium‐Based Metal‐Organic Frameworks as Novel Sorbents to Improve the Efficacy of Hemodialysis Treatment

**DOI:** 10.1002/smsc.202500054

**Published:** 2025-05-08

**Authors:** Fátima Guerrero, Francisco G. Moscoso, Joaquín Silvestre‐Albero, Alejandro Martin‐Malo, Carolina Carrillo‐Carrión

**Affiliations:** ^1^ Maimonides Biomedical Research Institute of Cordoba (IMIBIC) University of Córdoba 14004 Córdoba Spain; ^2^ Redes de Investigación Cooperativa Orientadas a Resultados en Salud (RICORS) Instituto de Salud Carlos III 28029 Madrid Spain; ^3^ Center for Nanoscience and Sustainable Technologies (CNATS) Departamento de Sistemas Físicos, Químicos y Naturales Universidad Pablo de Olavide 41013 Sevilla Spain; ^4^ Laboratorio de Materiales Avanzados Departamento de Química Inorgánica‐Instituto Universitario de Materiales Universidad de Alicante 03690 San Vicente del Raspeig Spain; ^5^ Institute for Chemical Research (IIQ) CSIC‐University of Seville Avda. Américo Vespucio 49 41092 Sevilla Spain

**Keywords:** dialysis, metal‐organic frameworks, perfluoroalkyl‐ligands, protein‐bound uremic toxins, uremic toxins

## Abstract

People with end‐stage chronic kidney disease (CKD) require routine dialysis treatments to remove uremic toxins from the blood in order to minimize toxic symptoms. However, some hydrophobic toxins cannot be effectively removed using conventional hemodialysis techniques, especially when they are bound to plasma proteins. This work reports the first experimental evidence addressing this issue through the incorporation of perfluoroligands into a zirconium‐based metal‐organic framework, taking advantage of the favorable interactions between fluorine atoms and albumin proteins. The as‐designed fluorinated NU‐1000 particles (NU@F) demonstrate their capability to remove not only free hydrophobic uremic toxins, specifically p‐cresyl sulfate (pCS) and indoxyl sulfate (IS), but also a large fraction of those bound to human serum albumin, notably without causing significant hypoalbuminemia. The NU@F particles incorporated into a cartridge also exhibit good performance under dynamic flow conditions, mimicking the real scenario of hemodialysis. Finally, the NU@F dialysis system is tested in a pooled sample from CKD patients, confirming the actual application potential of the developed prototype system.

## Graphical abstract

The incorporation of perfluoroalkyl chains into porous NU‐1000 leads to superior performance in the removal of uremic toxins from samples of CKD patients for two main reasons: improved stability and enhanced removal efficiency of protein‐bound toxins. The dialysis approach based on fluorinated NU‐1000 simultaneously exploits adsorption‐based and displacement‐based mechanisms and is compatible with flow systems.

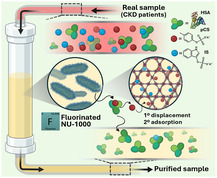

## 1 Introduction

Patients with chronic kidney disease (CKD), a condition affecting ≈13% of the general population worldwide, tend to accumulate uremic toxins in blood, increasing the circulating levels of these toxins with the progression of renal failure.^[^ ^1^ ^]^ Diabetes and high blood pressure are the two main causes of CKD, which together account for two‐thirds of cases; however, other diseases can also lead to kidney failure, including for example glomerulonephritis and polycystic kidney disease.^[^ ^2^, ^3^ ^]^ Moreover, the progression of CKD to its advanced stages inevitably leads to the appearance of endothelial dysfunction, overproduction of reactive oxygen species, persistent inflammation, and cardiovascular complications,^[^ ^4^, ^5^ ^]^ which are recognized as critical conditions perpetuating the bidirectional vicious cycle between CKD and systemic complications. These facts not only underline the scale of the problem but also emphasize the need to develop more efficient systems for removing uremic toxins from the blood, in order to face a wide range of pathological conditions.

Among the different types of uremic toxins, the most problematic are those with low molecular weight, hydrophobic character (i.e., aromatic fraction) and charged (i.e., ionic functional group), such as p‐cresyl sulfate (pCS) and indoxyl sulfate (IS), because they are primarily bound to the most abundant plasma protein, namely, human serum albumin (HSA), with high binding affinities to Sudlow's site II on HSA.^[^ ^6^ ^]^ Protein‐bound uremic toxins (PBUTs) are poorly removed by conventional hemodialysis (HD) technique, due to their high plasma protein binding, and consequent low, free (dialysable) plasma concentration. When HSA is present, the fraction of free pCS and IS available to diffusion through the membrane in the dialyzer is estimated as low as 2%–5%.^[^ ^7^ ^]^ The removal of PBUTs is still a major challenge. Despite the advances in dialysis treatments, the removal rate of total pCS and IS (both protein‐bound and unbound) hardly reaches values of 50% even when using synthetic high‐flux membranes.^[^ ^8^ ^]^ To date, the use of sorbents in hemoperfusion or plasma perfusion has proven to be the most effective strategy to increase the removal rate of toxins. This behavior is attributed to the capacity of adsorption‐based HD to exploit the intrinsic properties of sorbents for binding toxins based on chemical affinity, rather than relying simply on membrane permeability, molecular weight cut‐off, and sieving coefficient as the physical mechanisms behind diffusion and convention processes. Alternatively, displacement‐based HD is also being investigated, where several drugs (ibuprofen, furosemide, or phytodrugs)^[^ ^9^, ^10^, ^11^ ^]^ have been reported as effective displacers (i.e., albumin‐binding competitors). However, the intrinsic toxicity of these displacers must be taken into account when considering them for long‐term use in routine dialysis treatments. With this in mind, the combination of adsorption‐based and displacement‐based HD, as we intend to explore in this work, could open a new approach in the search for more effective and versatile dialysis systems.

Within this context, metal‐organic frameworks (MOFs), porous materials constructed by organic linker molecules coordinated to metal nodes in a repeating array,^[^ ^12^ ^]^ are considered promising sorbents to implement dialysis systems in order to specifically remove target toxins.^[^ ^13^, ^14^, ^15^, ^16^ ^]^ Unlike other porous materials (e.g., zeolites and carbons), MOFs are more versatile and advantageous as high‐capacity sorbents due to the larger surface areas per unit volume and higher structural and functional tunability (i.e., adjustable pore size, tunable hydrophilic/hydrophobic nature, and functionalization with specific chemical groups for selective interactions). In a previous work, our group demonstrated that zeolitic‐imidazolate framework‐8 (ZIF‐8) nanoparticles were able to remove quite efficiently two target uremic toxins (p‐cresol and indoxyl sulfate) in its free form, even after being immobilized within a renal scaffold and under flow operating conditions.^[^ ^17^ ^]^ The surface functionalization of the ZIF‐8 with a polymer was found to be key to providing stability within the scaffold microenvironment and establishing strong interactions with the extracellular matrix of an acellular kidney scaffold. The particular features of ZIF‐8, specifically its nanosize, low cytotoxicity and high biocompatibility made it the ideal candidate for the future development of “artificial kidneys”. However, when it comes to using MOFs in adsorption‐based dialysis systems, more robust types of MOFs are desirable. In this regard, Farha et al. selected zirconium‐based MOFs due to their generally remarkable water and chemical stability,^[^ ^18^ ^]^ and compared the performance of several Zr_6_‐based MOFs (e.g., UiO‐66, UiO‐67, MOF‐808, NU‐1000, NU‐1010, NU‐1200) to adsorb the toxin pCS from aqueous solution.^[^ ^13^ ^]^ The NU‐1000, composed of zirconium nodes connected by a pyrene‐based linker, led to the highest toxin removal efficiency with a value of 156.7 nmol of pCS per mg NU‐1000 after incubation in static conditions for 24 h at room temperature. Importantly, other Zr‐based MOFs with comparable surface areas and pore sizes to NU‐1000, but lacking an extended aromatic system, exhibited much lower removal efficiency of pCS.^[^ ^13^, ^16^ ^]^ This was attributed to favorable interactions between pCS and the hydrophobic adsorption sites formed by two pyrene linkers (*π*–*π* interactions), along with hydrogen bonding between the ionic sulfate groups of pCS and the hydroxyl groups on Zr_6_‐nodes.^[^ ^13^, ^14^ ^]^

Despite the promising results achieved so far, two critical drawbacks require further investigation for a potential application of NU‐1000 in adsorption‐based HD, i.e., 1) the limited stability of NU‐1000 in the presence of Lewis bases (e.g., phosphate and sulfate species)^[^ ^19^ ^]^ —noting that recyclability is an important feature of MOFs to be considered for dialysis techniques, and 2) the still challenge of improving the adsorption capacity of PBUTs, but without the associated risk of significant hypoalbuminemia. In order to solve these issues, fluorination of NU‐1000 could actually be an appealing strategy. On the one hand, the incorporation of fluorine groups into Zr‐based,^[^ ^20^, ^21^ ^]^ Cu‐based,^[^ ^22^ ^]^ and Zn‐based^[^ ^23^ ^]^ MOFs has already been demonstrated to protect the framework not only from the attack of water molecules but also from other chemical species such as phosphate anions. Moreover, fluorine centers allow for selective interactions, which have been exploited in the adsorption of CO_2_,^[^ ^24^, ^25^, ^26^ ^]^ SO_2_,^[^ ^27^ ^]^ and olefin/paraffin separations.^[^ ^28^ ^]^ In particular, NU‐1000 functionalized with various fluorine‐containing ligands via a solvent‐assisted ligand incorporation (SALI) approach has been explored both experimentally and theoretically for CO_2_ capture.^[^ ^26^ ^]^ On the other hand, recent studies have reported the ability of perfluoroalkyl substances (PFAS) to strongly interact with proteins, mainly albumins such as HSA and bovine serum albumin (BSA).^[^ ^29^, ^30^, ^31^, ^32^ ^]^ These works revealed that these interactions occurs via both specific binding sites within the hydrophobic pockets and nonspecific adsorption on the protein surface. In fact, this property has been smartly exploited for PFAS removal from environmental samples using proteins (BSA and lysozyme) as efficient adsorbents.^[^ ^33^ ^]^ However, to the best of our knowledge, neither PFAS nor perfluoroalkyl ligands —either free or grafted onto MOFs—have been explored in the context of dialysis applications, or as potential albumin‐binding competitors to increase the free fraction of toxins by displacement from protein‐toxin complexes.

Going one step further in the idea of using MOFs in adsorption‐based HD systems, it is important to highlight that dialysis operates under dynamic flow conditions. Most studies reporting MOFs for uremic toxins adsorption have been carried out in closed vials under static conditions, which is quite far from realistic conditions. It is well known when working with MOFs, that the translation from static conditions (in batch) to dynamic conditions (in flow) is not as straightforward as one might think, as specific requirements must be met—particularly regarding particle size (ideally nano‐ or submicron) and colloidal stability (i.e., lack of aggregation in suspension).

With these challenges in mind, the focus of this work is twofold: 1) to study the impact of fluorination of NU‐1000 on the removal of two critical uremic toxins (pCS and IS), in both free‐ and protein‐bound forms, aiming to combine the good properties of NU‐1000 for the elimination of free uremic toxins (i.e., adsorption‐based HD) with the potential of perfluoroalkyl‐ligands to bind HSA and serve as displacers for PBUTs (i.e., displacement‐based HD); and 2) to develop a MOF cartridge prototype for evaluating its performance in a dialysis setup under flow conditions. Important aspects related to stability, reusability, potential undesired albumin loss, and possible cytotoxicity of the dialysate/ultrafiltrate after passing through the MOF cartridge will be carefully assessed.

## 2 Results and Discussion

**Figure 1:**
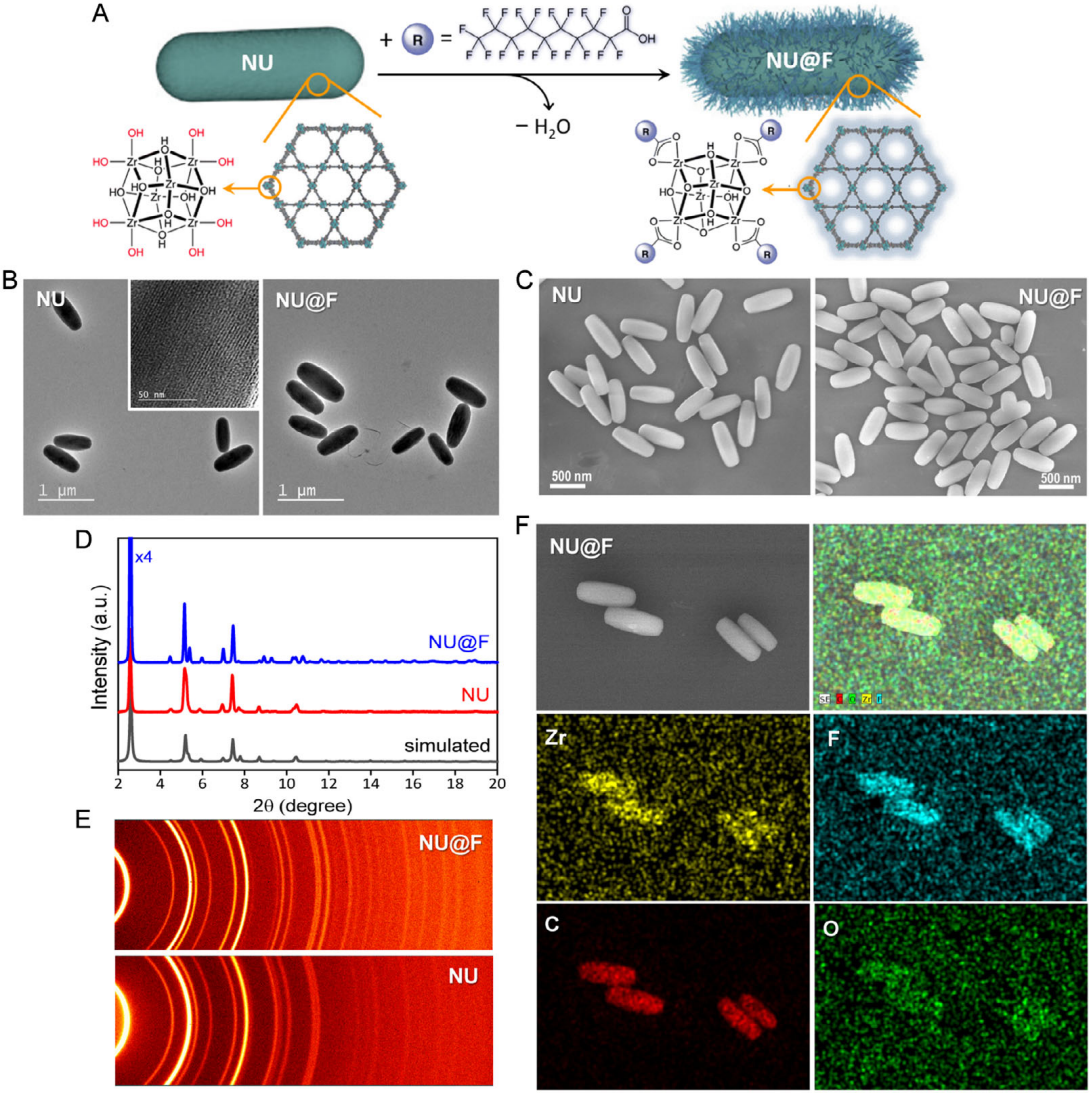
A) Schematic representation of the SALI functionalization of NU‐1000 (denoted as NU) with PFDA (C_9,F_) to afford the fluorinated NU‐100 particles (denoted as NU@F). B) TEM images of the NU and NU@F particles. Inset: HRTEM of a NU‐1000 particle showing the oriented mesoporous 1D channels. C) SEM images of the NU and NU@F particles. D) PXRD spectra of the NU and NU@F particles and the simulated pattern for comparison. E) 2DXRD scan of NU and NU@F particles. F) SEM‐EDX elemental mapping, showing the homogeneous distribution of the elements (Zr, F, C, and O) in the functionalized NU@F particles.

We succeeded in preparing high‐quality, phase‐pure crystalline, and submicron‐sized NU‐1000 particles, with the formula [Zr_6_(μ_3_‐O)_4_(μ_3_‐OH)_4_(H_2_O)_4_(OH)_4_(TBAPy)_2_]_*n*_ [TBAPy = 1,3,6,8‐(p‐benzoate)pyrene], which were further functionalized with perfluoroalkyl‐ligands (specifically a perfluoroalkyl carboxylic acid) using a solvent‐assisted ligand incorporation (SALI) approach (**Figure** **1**A). First, NU‐1000 was synthetized following a reported protocol,^[^ ^34^ ^]^ but with some modifications aimed at decreasing the particle size to the submicron scale. This was pursued for two main reasons: 1) the smaller the particle size, the lesser is the diffusion barrier for analytes, thus enhancing interaction between the adsorbate molecules and the adsorption sites within the MOF, and 2) smaller MOF particles tend to form more stable colloids, which helps prevent sedimentation in the dialysis setup, thus improving performance under flow conditions. Briefly, NU‐1000 particles with an average length of ≈700 nm were prepared under solvothermal conditions by mixing preformed Zr_6_ clusters and H_4_TBAPy in dimethylformamide (DMF), and using trifluoroacetic acid (TFA) as a modulator agent. The role of TFA was to eliminate the NU‐901 phase formation during the early stage of crystallization, as previously reported by Farha et al.^[^ ^34^ ^]^ We found that precise control of the heating temperature (100 °C) and continuous gentle stirring (400 rpm) were critical for controlling the kinetics of nucleation and crystal growth, resulting in highly homogeneous rod‐shaped particles of ≈700 nm in length within just 1 h of reaction, while maintaining a good yield (≈420 mg of activated NU‐1000). Details of the synthesis and activation procedures for NU‐1000 (denoted from now on as NU particles for simplicity) are given in the Supporting Information (SI), along with the crystal structure of NU‐1000 viewed from different orientations (Figure S1, Supporting Information). Scanning and transmission electron microscopy (SEM and TEM; Figure 1B,C and S2, Supporting Information) clearly showed the uniform size and shape of the as‐prepared NU particles. Closer inspection by high‐resolution TEM (HRTEM; inset in Figure 1B) showed the 1D mesoporous hexagonal channels of the framework. Besides, the powder X‐ray diffraction (PXRD) spectrum and 2D XRD scan (2DXRD) revealed the high crystallinity of particles (Figure 1D,E), consistent with the simulated XRD pattern and confirming the phase purity of NU‐1000. Fourier‐transform infrared spectroscopy (FT‐IR) provided evidence of coordination between the Zr_6_ clusters and the carboxylate groups of H_4_TBAPy ligands, showing the characteristic vibrational bands of NU‐1000 as expected (Figure S3, Supporting Information). In a second step, we performed the functionalization of NU‐1000 with perfluorodecanoic acid (PFDA) via the SALI approach, which allowed the attachment of the carboxylic acid of ligands to the free and exposed hydroxyl‐groups from the Zr_6_ nodes (Figure 1A). This post‐synthetic modification introduces perfluoroalkyl‐groups as charge‐compensating moieties strongly bound to the Zr_6_ node via ionic bonding.^[^ ^35^ ^]^ Among the available perfluoroalkyl carboxylic acids, we selected PFDA (C_9,F_) for its optimal interaction with the HSA protein, based on previous studies.^[^ ^32^ ^]^ These works, which used equilibrium dialysis to directly assess PFAS‐protein binding (with BSA as a model albumin protein), found that the fraction of bound PFAS increased with the number of fluorinated carbons (from C_4,F_ to C_9,F_). Moreover, PFDA (C_9,F_) exhibited a remarkable binding cooperativity effect, as determined using the Hill adsorption model.^[^ ^36^ ^]^ To carry out the functionalization, we followed a reported procedure^[^ ^26^ ^]^ that consisted in incubating the as‐prepared NU‐1000 particles with a PFDA solution (in a ratio of 8 equivalents per Zr_6_ node) in DMF at 60 °C for 24 h (see SI for details). The resulting fluorinated particles (denoted from now on as NU@F for simplicity) preserved their morphology, as observed by SEM and TEM (Figure 1A,B and S2, Supporting Information). ^1^H and ^19^F nuclear magnetic resonance (NMR) spectra of the NU@F after decomposing the particles in a 10% D_2_SO_4_/DMSO‐d6 mixture (Figure S4, Supporting Information) confirmed the successful functionalization, while elemental mapping using energy‐dispersive X‐ray spectroscopy (EDX) revealed a homogeneous distribution of the fluorine atoms throughout the particles (Figure 1F and S5, Supporting Information). Regarding the crystallinity, the PXRD spectrum of NU@F showed sharp peaks at low angles, indicating that the high crystallinity of the particles was retained after functionalization (Figure 1D). Note that the change observed in the relative peaks intensities upon functionalization, specifically the increase in the peak at a 2*θ* value of 2.5, suggests an increased electron density around the (010) plane, where a significant portion of the Zr_6_ node is located. This observation supports that functionalization occurred at the Zr_6_ nodes.^[^ ^26^ ^]^ The preservation of crystallinity upon functionalization was more clearly evidenced by the more sensitive 2DXRD technique, which displayed strong and well‐defined Debye rings with uniform intensity distribution (Figure 1E) and also showed changes in relative peaks intensities. The Miller indices and d‐spacing values of the reflection peaks are listed in Table S1 (Supporting Information).

**Figure 2:**
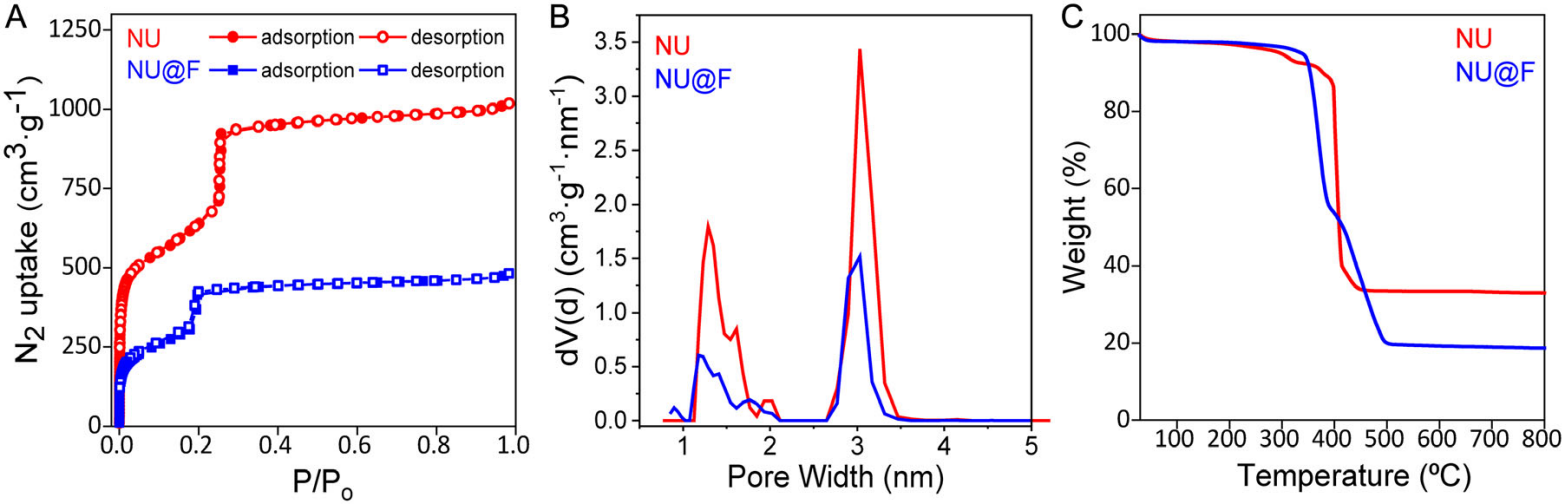
A) N_2_ isotherms (77 K) of the as‐prepared NU and NU@F particles. B) The corresponding NLDFT pore size distribution analyses, showing changes on both the micropores and mesopores upon functionalization. C) TGA profiles of the NU and NU@F particles in a dynamic air atmosphere.

Next, we studied the changes in the vibrational modes of the bonds after perfluoro‐functionalization of the NU particles using FTIR spectroscopy. As shown in Figure S3 (Supporting Information), there was a significant increase in the band at 1411 cm^−1^ in the NU@F spectrum compared to that of pristine NU particles, which is attributed to the carboxylate groups from the perfluoroalkyl‐ligands coordinated to the Zr_6_ clusters. Furthermore, new bands appeared in the range 1120–1260 cm^−1^, associated to symmetric and asymmetric stretching vibrational modes of the —CF_2_‐ groups of the perfluoroalkyl‐chains. These observations further confirmed the successful functionalization of the NU particles without altering their primary framework, as the characteristic FT‐IR bands of the structure remained essentially unchanged. Subsequently, we evaluated the impact of incorporating fluoroalkyl‐groups on the textural properties of NU‐1000 by measuring the N_2_ isotherms of the as‐prepared NU and NU@F particles. As shown in **Figure** **2**A, both samples presented a combination of type I and type IV isotherms, according to IUPAC classification, with two steep increases at the relative pressures of *P*/*P*_o_ ≈ 0.01 and ≈0.25, indicating the presence of both micropores and perfectly defined mesopores. In the functionalized sample, the mesoporous step shifted slightly to a lower pressure, from *P*/*P*_o_ = 0.25 for NU to *P*/*P*_o_ = 0.2 for NU@F, indicating a reduction in mesopore size upon fluoroalkyl‐groups incorporation (Table S2, Supporting Information). As expected, functionalization led to a notable decrease in surface area (S_BET_), which dropped from 2235 m^2^ g^−1^ for NU to 1097 m^2^ g^−1^ for NU@F, along with a 47% reduction in total pore volume (Table S2, Supporting Information). In addition, the average mesopore size decreased to 2.9 nm in NU@F compared to 3.1 nm in NU, as determined from pore size distribution obtained after application of the nonlocal density functional theory (NLDFT) to the N_2_ adsorption data (Figure 2B and S6, Supporting Information). Note that the estimated micro‐ and mesopore sizes in NU particles (1.3 and 3.1 nm, respectively) fitted quite well with the theoretical values for NU‐1000 (1.3 and 3.3 nm, respectively). These results further confirmed the successful functionalization of NU‐1000 and indicated that the perfluoroalkyl‐groups were attached at the Zr_6_ nodes located within the internal cavities (filling the mesopores), rather than being exclusively attached to  external nodes on the particles surface. Thermogravimetric analyses (TGA) in air showed that NU particles remained stable up to 400 °C, with a rapid weight loss above this temperature due to collapse of the NU‐1000 framework and descomposition of the organic ligands. In contrast, NU@F particles presented a more gradual decomposition of the framework within the range 340–500 °C (Figure 2C). Differential scanning calorimetry (DSC) curves further revealed that the exothermic peak in NU@F became broader, suggesting the presence of multiple overlapping thermal events (Figure S7, Supporting Information). Notably, NU@F had a residual ash content of 18.7 wt%, compared to 33.0 wt% for the non‐functionalized NU particles. This difference of 14.3 wt% in the mass fraction of the inorganic residue (corresponding to ZrO_2_) reflects the amount of incorporated perfluoroalkyl‐ligands.

**Figure 3:**
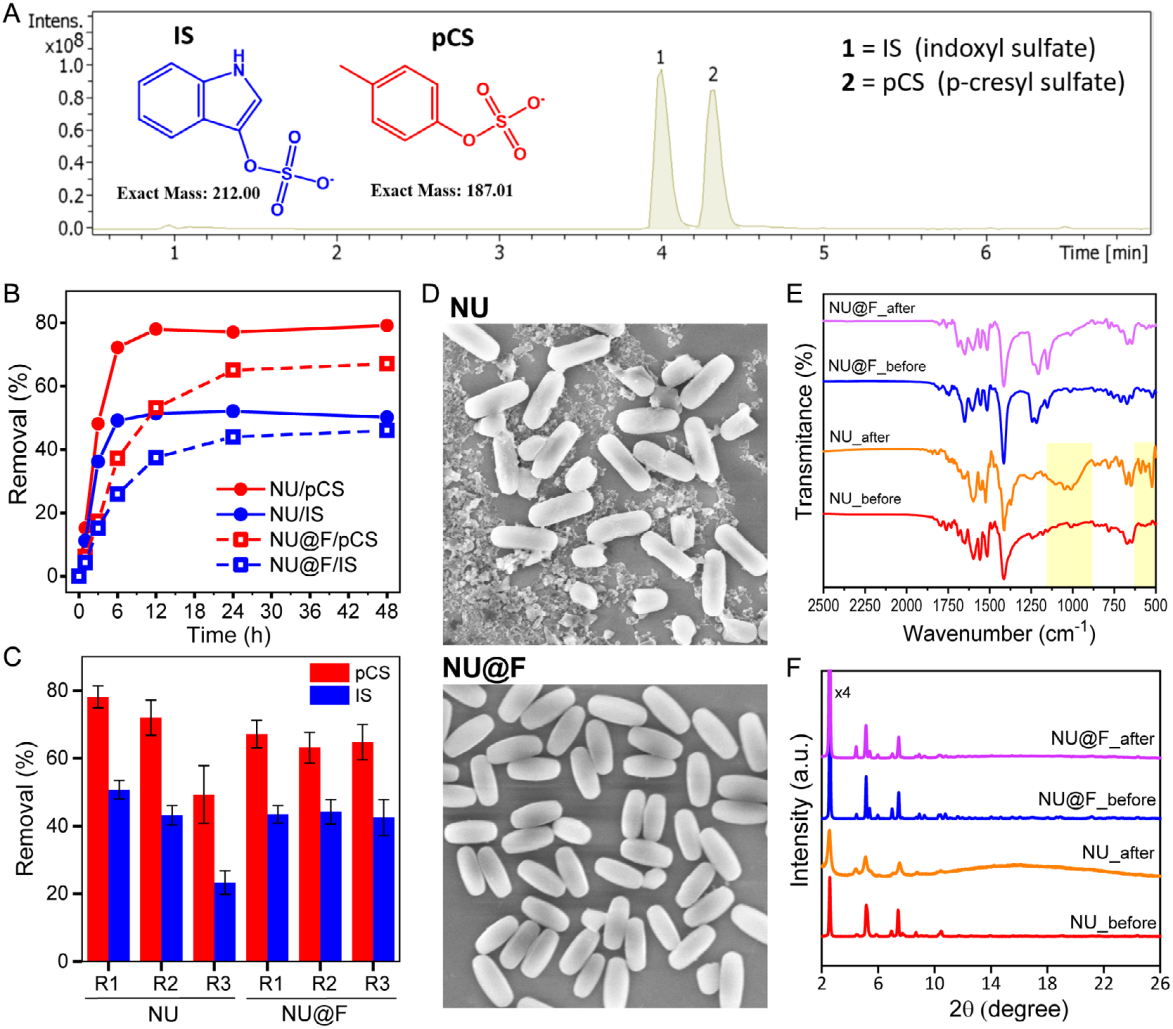
A) Representative HPLC chromatogram of a mixture of both toxins in PBS (pCS and IS, 0.3 mM each). The chemical structures of toxins are shown. B) Removal efficiencies as a function of time at RT (22 °C) of NU and NU@F particles in a solution containing both uremic toxins (pCS and IS, 0.3 mM each) in PBS (10 mM, pH = 7.4). C) Removal efficiencies of NU and NU@F particles after 24 h incubation in three successive adsorption experiments (R1, R2, and R3); conditions: 6.5 mL of 0.3 mM pC and IS in PBS (10 mM, pH = 7.4) and 10 mg of MOF. Data are expressed as mean value ± SD of three independent experiments. After each use, particles were collected by centrifugation and regenerated (see SI for details). D) SEM images of NU and NU@F particles recovered after the third adsorption experiment. E) FTIR spectra of the NU and NU@F particles before use and after the third reuse. The observed changes in the NU particles are marked in yellow. F) PXRD spectra of the NU and NU@F particles before use and after the third reuse.

Upon evaluation of the physicochemical properties of both NU and NU@F particles, we investigated their capacity to remove two common uremic toxins (pCS and IS) under static conditions by incubating the particles (10 mg) with a mixture of both toxins (0.3 mM each, in PBS). These toxins were selected due to their problematic nature, as they exhibit high binding affinity to plasmatic proteins, and thereby cannot be efficiently removed by conventional HD, as mentioned above. The toxins concentration was set at 0.3 mM each to simulate the blood of CKD patients under worst‐case conditions (i.e., levels found in end‐stage renal disease).^[^ ^37^ ^]^ Quantification of the toxins was carried out by reversed‐phase high‐performance liquid chromatography (HPLC) analysis as shown in **Figure** **3**A (see SI for details; Figure S8–S10 and Table S3, S4, Supporting Information). To initially assess the effect of the perfluoroalkyl‐chains on the adsorption process, we performed kinetic studies by varying the incubation time of the adsorption experiments (6.5 mL of a PBS solution containing both toxins, 0.3 mM each toxin, 10 mg MOF, incubation at RT ≈ 22 °C). As shown in Figure 3B, the pristine NU particles presented a significantly faster adsorption of both toxins compared to the NU@F particles—an expected result due to diffusion limitations caused by the long perfluoroalkyl‐chains. However, the adsorption efficiencies at saturation were quite similar for both particles (pristine and functionalized), with notably higher uptake for pCS compared to IS (Figure 3B, Table S5, Supporting Information). It is important to note that experiments were performed with 10 mg of MOF particles, but in the case of the functionalized NU@F particles, the actual MOF content is notably lower, considering a 14.3 wt% of perfluoroalkyl‐ligands as determined by TGA. Therefore, comparison expressed as nmol of toxin adsorbed per mg of NU fraction is given in Table S5, Supporting Information, showing uptake values of ≈150 mmol mg^−1^ for pCS and ≈100 mmol mg^−1^ for IS under our experimental conditions. The fact that the perfluoroalkyl‐functionalization does not result in any appreciable change in the maximum uptake capacity (at saturation) of these toxins, assuming that the perfluoroalkyl‐chains are attached to the hydroxyl groups on Zr_6_ nodes, suggests that *π*–*π* interactions with the pyrene linkers are the main driving force behind the adsorption process, whereas potential hydrogen bonding between hydroxyl groups on Zr_6_ with the ionic sulfate groups of the toxins does not appear to play a determining role. In parallel, to compare the performance of our as‐synthetized NU particles with those prepared by Kato et al.^[^ ^13^ ^]^ using the same MOF type (i.e., NU‐1000), we conducted an adsorption experiment under identical experimental conditions to that reported, i.e., individual pCS adsorption assay, using a 0.1 mM aqueous pCS solution, 1.5 mg of MOF, RT, and 24 h (Table S5, Supporting Information). Under these conditions, our NU removed 178 nmol mg^−1^ pCS, a notably higher uptake than the reported value of 156 nmol mg^−1^. This difference may be attributed to structural differences between the materials, most likely related to the effect of the particle size. Note that our NU particles are significantly smaller (0.7 vs. 10 μm), remaining colloidally stable in the toxins solution (Figure S11, Supporting Information), which likely facilitates diffusion and access of pCS molecules to the internal pores (i.e., less accessible adsorption sites) within the framework.

Next, in an attempt to reuse the MOF particles, we found that the removal efficiencies decreased significantly after the third use in the case of pristine NU particles, while gratifyingly, NU@F perfectly reproduced its adsorption behavior in successive uses (Figure 3C; Table S5, Supporting Information). This can be explained by the increased chemical stability of the NU‐1000 framework upon functionalization. It is well known that the stability of NU‐1000, like that of other Zr‐carboxylate MOFs, is compromised by the presence of phosphate ions,^[^ ^19^ ^]^ which act as ligands due to their Lewis base character, replacing the organic ligands in the MOF and promoting the formation of surface defects, ultimately leading to particle dissolution. Likewise, sulfate species can also attack open metal sites on the Zr_6_ nodes through Zr–sulfate coordination,^[^ ^38^, ^39^ ^]^ resulting in defective frameworks that are more prone to collapse during regeneration steps. This means that in the presence of phosphate and/or sulfate species (e.g., sulfate uremic toxins such as pCS and IS), especially when present at high concentrations, NU‐1000 is very susceptible to gradual degradation over time, losing its ability to remove these toxins. In fact, this is precisely what we observed in the NU particles when working with high concentrations of uremic toxins (0.3 mM each) in PBS solution, a 1.6‐fold and 2‐fold decrease in the removal efficiency after the third use for pCS and IS, respectively. Notably, this limitation of pristine NU‐1000 was not reported in previous studies by other researchers,^[^ ^13^, ^15^ ^]^ likely because those adsorption experiments used toxin solutions at 0.1 mM in pure water, and because the NU‐1000 particles used were too large (≈10 μm)^[^ ^34^ ^]^ to exhibit observable degradation. However, it is important to remark here that phosphate is an essential electrolyte in the human body, constituting about 1% of total body weight. Moreover, hyperphosphatemia occurs in advanced stages of renal impairment, with serum phosphate concentrations >5.5 mg dL^−1^ found in CKD patients.^[^ ^40^ ^]^ Thus, the potential presence of significant quantities of phosphate ions in patients undergoing hemodialysis must be taken into account when designing adsorbents for uremic toxins removal.

To study in detail the structural changes of NU and NU@F particles after the adsorption experiments, we performed SEM, PXRD, and FTIR analyses on the recovered particles after the third use. As shown in Figure 3D, degradation of NU particles was clearly visible under the microscope, while NU@F preserved its morphology without notable changes; more representative images are provided in Figure S12, Supporting Information. The FTIR spectra (Figure 3E) of reused particles confirmed the attack of the phosphate ions to the framework in the case of NU particles, showing the appearance of new bands in the range of 500–600 cm^−1^, attributed to O—P—O bending vibrations, and a broad band at 1000 cm^−1^, arising from P—O stretching vibrations.^[^ ^41^ ^]^ In contrast, the perfluoroalkyl‐chains in the NU@F particles protected the framework from such attack, as no evidence of phosphate incorporation in the structure was observed in the FTIR spectrum. To quantify the impact on crystallinity (Figure 3F), we determined the crystalline index (CI; see details in SI) of the particles before and after adsorption experiments. The CI of reused NU particles decreased significantly, dropping to 45% from the initial value of 85% before use, confirming the generation of numerous crystalline defects as a consequence of phosphate attack. Again, NU@F results demonstrated that perfluoro‐functionalization is beneficial for stabilizing the framework and retaining its crystallinity, with CI values of 75% and 72% before and after use, respectively. It is worth noting that the slightly lower CI of NU@F (75%) compared to pristine NU particles (85%) is likely due to the contribution of the non‐crystalline fraction from the long fluorinated alkyl chains (14.3 wt% according to TGA) to the total particle mass.

**Figure 4:**
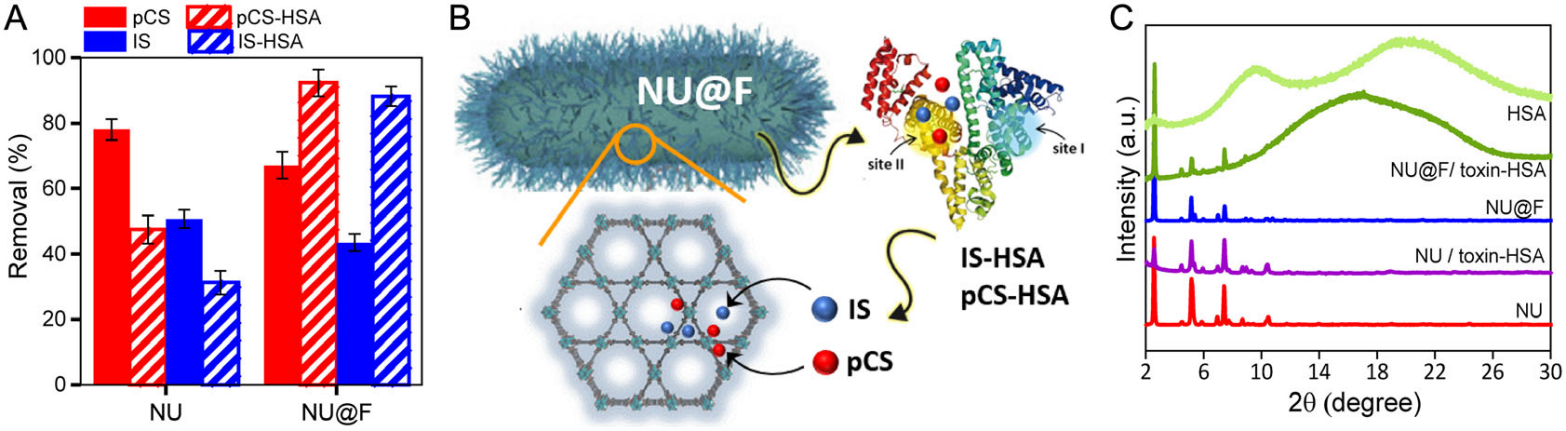
A) Removal efficiencies of NU and NU@F particles toward either free toxins (pCS and IS) or protein‐bound toxins (pCS‐HSA and IS‐HSA complexes); conditions: 6.5 mL of toxin‐HSA solution (preincubated 0.3 mM pCS + 0.3 mM IS + 0.6 mM HSA for 24 h at RT in PBS), 10 mg MOF, and incubation for 24 h at RT. B) Schematic illustration of the dual role of the NU@F particles, (i) as displacers of the toxins from the toxin‐HSA complexes and (ii) as adsorbents for removing the free toxins. C) PXRD spectra of the NU and NU@F particles after incubation with toxin‐HSA complexes.

Going a step further, and bearing in mind that the key challenge with the studied toxins (pCS and IS) is their high affinity to proteins, we investigated the capacity of both NU and NU@F particles to remove pCS and IS from toxin‐protein complexes, specifically pCS‐HSA and IS‐HSA. Note that HSA is the major albumin protein present in human blood, with HSA concentrations in plasma ranging from 35 to 50 mg mL^−1^ (i.e., 0.52–0.75 mM).^[^ ^42^ ^]^ To this end, toxins (pCS + IS, 0.3 mM each) were first pre‐incubated with 0.6 mM HSA for 24 h at RT in PBS solution to allow the formation of toxin‐HSA complexes. Afterwards, this toxin‐HSA mixture was subjected to adsorption experiments using either NU or NU@F particles (Table S6, Supporting Information). It is important to note that the use of equimolar amounts of toxins and HSA ensures that all toxin molecules are potentially forming toxin‐HSA complexes. As shown in **Figure** **4**A, in the case of NU particles (i.e., pristine NU‐1000), we observed a notable decrease in the removal efficiency of both toxins when using the preformed toxin‐HSA complexes. Increasing the amount of NU particles (from 10 to 15 mg) while keeping the toxin‐HSA concentration constant did not lead to any significant improvement in the removal efficiency. This seems to indicate that the fraction of free toxins is already quantitatively removed with 10 mg of particles, but the fraction of toxins bound to HSA (likely strongly attached to Sudlow's site II in HSA)^[^ ^6^ ^]^ cannot be detached from the protein under the tested experimental conditions (static incubation at 22 °C), thus preventing further adsorption within the pores of the MOF. Our findings differ from those reported by Kato et al.^[^ ^13^ ^]^ who achieved almost complete removal (>90%) of pCS by NU‐1000 simply by increasing the amount of MOFs (from 6 mg in the absence of HSA to 20 mg in the presence of HSA). Nevertheless, this implies that the adsorption capacity expressed as mmol mg^−1^ decreased significantly in the presence of HSA, making this material less efficient as an adsorbent under realistic dialysis conditions (i.e., samples containing plasmatic proteins). These discrepancies between our results and those reported^[^ ^13^ ^]^ could be attributed to two main factors. First, their experiments were conducted at 37 °C, a temperature at which toxin‐protein interactions are weaker and diffusion of free toxins is facilitated compared to RT (22 °C). Indeed, it has been reported that the percentage of protein‐bound toxins decreases as the temperature increases.^[^ ^43^ ^]^ Moreover, they used an aqueous solution of 0.2 M NaCl at pH 6.5, whereas we used a phosphate buffer solution at pH = 7.4 to better mimic physiological conditions, as normal blood pH ranges from 7.35 to 7.45. Second, the quantification of total toxins (free + protein‐bound) in experiments involving toxin‐HSA complexes requires a pretreatment step to denature and precipitate HSA, allowing the release of toxins from the complexes to be able to quantify them by HPLC analysis in a reliable way. Denaturation of HSA can be achieved by heat or by adding denaturing solvents such as methanol. Given that these toxins are sensitive to temperature (as indicated by commercial suppliers), we performed denaturation with methanol (see SI for details) as described previously,^[^ ^44^, ^45^ ^]^ instead of heating to 100 °C, as done by other researchers.^[^ ^13^ ^]^ It is obvious that such differences in sample analysis procedures can lead to discrepancies in the interpretation of MOF adsorption results, making this a critical aspect to consider in future studies.

Aiming to exploit the favorable interactions between the perfluoroalkyl‐chains in NU@F particles and HSA, as discussed above, we focused on evaluating the adsorption performance of these particles toward toxin‐HSA complexes. Importantly, the results changed significantly under this new scenario (Figure 4A), with NU@F achieving a 1.9‐fold and 2.8‐fold increase in removal efficiency for pCS and IS, respectively, compared to pristine NU particles. The greater improvement observed for IS by NU@F can be explained by differences in the binding affinities of the two toxins with HSA. IS has a higher binding affinity for HSA than pCS, as determined by isothermal titration calorimetry (*K*_a_ = 10.06 × 10^3^ M^−1^ for IS vs. *K*_a_ = 1.39 × 10^3^ M^−1^ for pCS).^[^ ^46^ ^]^ Thus, upon pre‐incubation of both toxins with HSA, the formation of IS‐HSA complexes will be favored over that of pCS‐HSA, likely resulting in a lower fraction of free IS available for adsorption by pristine NU particles. In contrast, the initially higher fraction of free pCS will make easier its adsorption on NU particles. Therefore, the benefit of using NU@F becomes especially pronounced for IS, where a larger fraction remains protein‐bound. Based on our findings, we hypothesize that the strong affinity of the perfluoroalkyl‐chains (present on the MOF particle surface) towards HSA facilitates the displacement of pCS and IS from their binding sites on the protein. Once released into the medium, the free toxins can reach easily the adsorption sites within the NU‐framework, as illustrated in Figure 4B. According to the literature, the binding constant of PFDA (the ligand attached to NU‐1000) to HSA is approximately *K*_a_ = 50 × 10^3^ M^−1^^[^ ^47^ ^]^ (or even higher depending on the experimental technique),^[^ ^48^ ^]^ values significantly higher than those of toxins‐HSA complexes, making displacement feasible. However, if complete displacement were occurring, the adsorption capacity of NU@F (expressed as mmol mg^−1^) for toxins in experiments with preformed toxin‐HSA complexes should be similar to that obtained in experiments using free toxins (i.e., in the absence of HSA), not substantially higher as we found in the case of NU@F particles (see Table S6, Supporting Information). This finding suggests that new adsorption sites for toxins have emerged in NU@F particles as a result of the functionalization—an aspect that will be carefully investigated in future studies.

To obtain further evidence of the favorable interaction between the functionalized NU@F particles and HSA, we measured the PXRD spectrum of the particles after being exposed to toxin‐HSA complexes. For comparison, we did the same with the non‐functionalized NU particles. As shown in Figure 4C, the diffraction peaks of NU@F remained visible (also in 2DXRD, Figure S13, Supporting Information), although an increase in the baseline and the appearance of a broad band, corresponding to the amorphous contribution of the HSA, rsulted in a slight decrease in overall crystallinity (CI decreased to 56%, Table S7, Supporting Information). HSA itself has a CI of 21%, reflecting its inherently amorphous nature. It is important to note that after washing the NU@F particles, HSA was easily desorbed from the particles, and their crystallinity was restored. In contrast, PXRD of the pristine NU particles did not show the characteristic protein band, indicating little to no interaction with NU‐1000, consistent with previous studies.^[^ ^13^ ^]^

**Figure 5:**
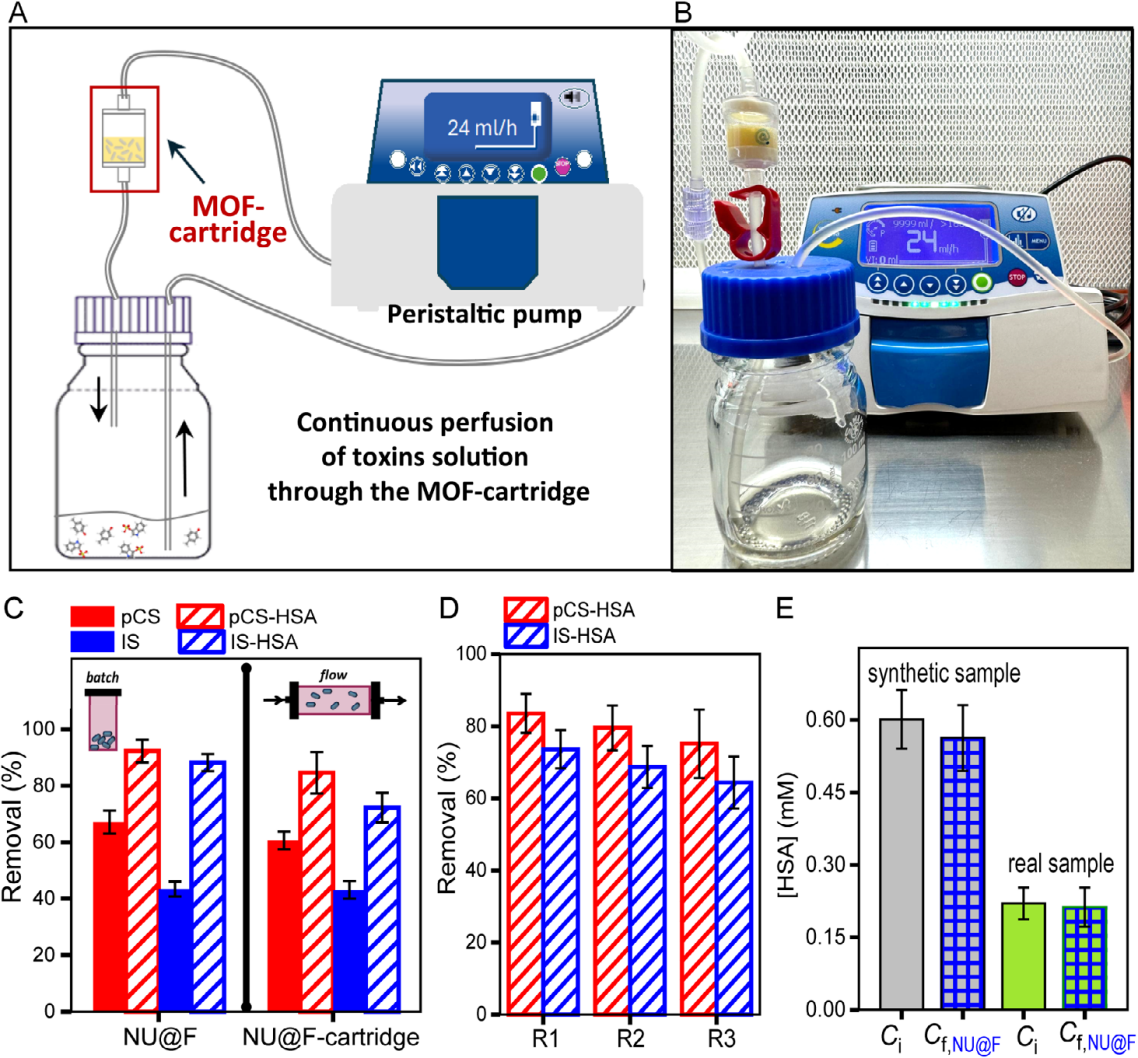
A) Scheme and B) photograph of the dialysis set‐up containing the MOF cartridge. C) Removal efficiencies after 24 h of the MOF cartridge in flow toward either free toxins (pCS and IS) or protein‐bound toxins (toxin‐HSA). Data obtained for the NU@F particles in batch are shown for comparison. D) Removal efficiencies of MOF cartridge in three successive uses (R1, R2, and R3). Data are expressed as mean value ± SD of three independent experiments. After each use, the MOF cartridge was regenerated (see SI for details). E) Concentration of HSA in the synthetic and real samples before (*C*_*i*_) and after being perfused through the MOF cartridge (C_f,NU@F_) for 24 h. Data are expressed as mean value ± SD of three independent experiments.

At this point, and in pursuit of a more realistic scenario, we proceeded to integrate NU@F particles into a dialysis device and evaluate their performance under flow conditions. We designed a dialysis setup as shown in **Figure** **5**A (see SI for details; Figure S14, Supporting Information), consisting of a closed circuit in which 20 mL of either solution of free toxins (0.3 mM pCS, 0.3 mM IS, in PBS) or solution of protein‐bound toxins (0.3 mM pCS, 0.3 mM IS, 0.6 mM HSA in PBS; mixture pre‐incubated 24 h at RT) was circulated using a peristaltic pump at a flow rate of 24 mL min^−1^ through the MOF cartridge (a reservoir containing 30 mg of NU@F particles dispersed in PBS) for 24 h. After this time, the concentration of toxins remaining in the perfused solution was quantified by HPLC and compared to the initial concentration to determine the removal percentages. A first positive observation was tha NU@F particles remained suspended in the reservoir as a homogeneous dispersion and were colloidally stable throughout toxin perfusion (see photograph in Figure 5B). Thanks to this colloidal stability under continuous flow, no overpressure issues ocurred—a common problem when particles settle and clog cartridge filters. Next, we evaluated the adsorption performance of the MOF cartridge for pCS and IS, both in their free and protein‐bound forms. Note that for technical reasons, these flow experiments were carried out with more amount of MOF particles (30 mg); therefore, the volume of the toxins solution was proportionally increased to maintain the same toxin:MOF ratio as in the batch experiments (i.e., static conditions). As shown in Figure 5C (Table S8, Supporting Information), the removal efficiencies in the perfusate (i.e., solution collected after passing through the MOF cartridge for 24 h) were comparable to those obtained under static conditions, again demonstrating enhanced removal of protein‐bound toxins with the functionalized NU@F particles.

Next, we investigated the potential reusability of the MOF cartridge for multiple cycles of toxins removal, particularly in the case of protein‐bound toxins. After testing several conditions, the optimal procedure to regenerate the NU@F particles inside the cartridge consisted of two sequential washing steps, first with methanol and then with PBS solution (see SI for details). Importantly, the decrease in the removal efficiency after each cycle was minimal (Figure 5D, Table S8, Supporting Information), which may be attributed to slight aggregation or minor particle degradation during washing. These results demonstrate that the MOF cartridge can be reused at least three times without a significant loss in toxins uptake capacity, following a relatively simple regeneration process.

On another note, the question may arise as to whether the perfluoroalkyl chains act as displacers or instead allow the adsorption of the toxin‐HSA complex as a whole. If the latter were the case, the amount of HSA in the sample (i.e., the solution containing toxin‐HSA complexes, prepared as described above) would be expected to decrease significantly after 24 h of perfusion. To explore this possibility, we quantified the amount of protein (see SI for determination procedure) in the initial sample and after perfusion through the MOF cartridge. No significant differences were observed (Figure 5E), indicating that the interaction between the perfluoroalkyl‐ligands on NU@F particles and HSA is dynamic under the experimental flow conditions, with HSA not being irreversibly adsorbed. This finding is crucial, as it minimizes the risk of causing hypoalbuminemia in patients undergoing dialysis—a common concern reported with some enhanced dialysis strategies such as super high‐flux hemodialysis membranes.^[^ ^49^ ^]^

Furthermore, to evaluate the feasibility of our MOF cartridge using real samples, we collected ultrafiltrate samples from CKD patients undergoing hemodialysis treatment (see SI for details). We analyzed this real sample by HPLC‐MS to quantify the initial toxins concentrations, which were found to be 0.023 and 0.012 mM for pCS and IS, respectively (Table S9, Supporting Information). Importantly, both toxins were completely removed after passing through the MOF cartridge, as shown in Figure S15 (Supporting Information). The sample was then fortified with an additional 0.3 mM of each toxin to mimic concentrations found in end‐stage CKD patients. In this case, toxins removal efficiencies were 75.1% and 65.0% for pCS and IS, respectively (Table S9, Supporting Information), which are comparable to the results obtained with synthetic samples. We also verified that the amount of plasmatic proteins in the real sample (initially 0.22 mM) was not significantly altered after 24 h of perfusion through the NU@F‐cartridge, ensuring no unwanted hypoalbuminemia (Figure 5E). It is worth highlighting the advantage of using MS detection in ESI negative ionization mode, which enables simultaneous and reliable detection of both toxins in biological real samples without interferences (i.e., a clean background), unlike the positive ionization mode, as shown in Figure S16 (Supporting Information). Additionally, coupling HPLC with MS detection rather than more typical detection techniques (such as DAD or fluorescence) is beneficial, as it provides unequivocal identification of the toxins, which is particularly important for real samples analysis.

Finally, we assessed the biosafety of the proposed MOF cartridge system by evaluating the in vitro cytotoxicity of perfused samples on endothelial cells after 24 h of treatment (see SI for details). To this end, HUVEC cells were exposed to increasing doses of the perfused sample, and we found that cell viability was not compromised at any dose (Figure S17, Supporting Information). This finding allows us to conclude that either no leaching of MOF particles (or their components) occurred during perfusion or, if it did, the amount was too small to cause toxicity. It is also worth notingo that zirconium is a biocompatible metal (human body contains around 300 mg of zirconium, with a recommended daily intake of 4.15 mg), making Zr‐based MOFs good candidates for biomedical applications.^[^ ^50^ ^]^

## 3 Conclusions

Altogether, the results obtained with the functionalized NU@F particles—namely, 1) their enhanced stability in the presence of Lewis bases, enabling potential reusability, and 2) their superior removal efficiency for protein‐bound toxins—clearly demonstrate the superior performance of perfluorinated NU‐1000 compared to its pristine counterpart for dialysis applications. Specifically, the innovative dialysis approach proposed in this work simultaneously leverages adsorption‐based and displacement‐based mechanisms. This dual functionality is enabled by the combination of the adsorption properties of NU‐1000 and the ability of the perfluoroalkyl‐ligands to displace uremic toxins bound to the major plasmatic protein (i.e., HSA). Importantly, NU@F maintained high removal efficiencies for both free and protein‐bound pCS and IS after integration into a cartridge, demonstrating for the first time the potential of MOFs for uremic toxins removal under flow conditions—thus mimicking the dynamic environment of hemodialysis. Moreover, the designed NU@F‐based dialysis treatment was successfully applied to real samples from CKD patients, a key step in validating the applicability of the method in real‐world scenarios.  In vitro toxicity studies with post‐treatment samples confirmed that the NU@F system meets biosafety criteria.

In conclusion, this work presents the first demonstration of the potential of fluorinated MOFs in the development of next‐generation dialysis devices, making progress toward improved treatment strategies for kidney failure patients. Beyond its relevance for kidney‐related diseases, the NU@F‐based dialysis system may also be extended to other pathologies that require blood purification therapies, for example, the removal of hydrophobic cytokines (molecules with chemical properties similar to PBUTs) to help mitigate systemic inflammatory response syndrome.^[^ ^51^ ^]^

## Conflict of Interest

The authors declare no conflict of interest.

## Author Contributions

**Fátima Guerrero**: formal analysis (equal); funding acquisition (equal); investigation (equal); methodology (equal); and writing—review and editing (equal). **Francisco G. Moscoso**: investigation (supporting) and writing—review and editing (equal). **Joaquín Silvestre‐Albero**: writing—review and editing (equal). **Alejandro Martin‐Malo**: funding acquisition (equal); methodology (supporting); and writing—review and editing (equal). **Carolina Carrillo‐Carrión**: conceptualization (lead); formal analysis (lead); funding acquisition (equal); investigation (lead); methodology (lead); supervision (lead); validation (lead); writing—original draft (lead); and writing—review and editing (lead).

## Supporting information

Supplementary Material

## Data Availability

The data that support the findings of this study are available in the supplementary material of this article.
