# Supplementary material for: Fluorinated Zirconium‐Based Metal‐Organic Frameworks as Novel Sorbents to Improve the Efficacy of Hemodialysis Treatment
Source: Small Sci. 2025 May 8;5(7):2500054. doi: 10.1002/smsc.202500054 (PMC12257890; doi:10.1002/smsc.202500054)
Supplement: Supplementary file 1 — Supplementary Material [file SMSC-5-2500054-s001.pdf]

Supporting Information

Fluorinated Zirconium-based Metal-Organic Frameworks as novel sorbents to improve the efficacy of hemodialysis treatment

*Fátima Guerrero, Francisco G. Moscoso, Joaquín Silvestre-Albero, Alejandro Martin-Malo, and Carolina Carrillo-Carrión**

TABLE OF CONTENTS	Page
**S1. General information**	2
**S2. Synthesis of NU-1000 particles (NU) and post-synthetic fluorination (NU@F)**	3
**S3. Morphological/structural characterization of NU and NU@F**	4
**S4. Quantification of toxins by HPLC-MS**	11
**S5. Adsorption experiments under static conditions**	14
**S6. Structural changes in NU and NU@F particles after toxins removal**	16
**S7. Adsorption experiments under flow using the dialysis setup**	18
**S8. Studies with human samples from patients**	20
**S9. Protein quantification and cell viability assays**	22

S1. General information

Chemicals: All reagents and solvents were commercially available and were used as received without further purification. The reagents used for the synthesis of MOFs were 4,4′,4″,4‴-(pyrene-1,3,6,8-tetrayl)tetrabenzoic acid (H4TBAPy; BLD Pharmatech; 97%), zirconyl chloride octahydrate ($ZrOCl_2 \cdot 8H_2O$; Sigma Aldrich; 98%), benzoic acid (BA; Sigma Aldrich; 99.5%), trifluoroacetic acid (TFA; Sigma Aldrich; ≥99%), perfluorodecanoic acid (PFDA; Sigma Aldrich; 98%), N,N-dimethylformamide anhydrous (DMF; Panreac, 99.8%), methanol (MeOH; Panreac, 99.9%). Uremic toxins, including p-cresyl sulfate potassium salt (pCS, BLD Pharmatech; 98%) and indoxyl sulfate potassium salt (IS, Sigma-Aldrich; 99%), were used as purchased without any purification. Human serum albumin (HSA, Sigma-Aldrich; lyophilized powder, ≥96%), was also used as received. PBS (0.01 M, pH=7.4) was prepared by dissolving PBS tablets (Sigma-Aldrich #P4417) in Milli-Q water.

Scanning Electron Microscopy (SEM): SEM images were taken using a HITACHI S4800 field emission microscope operating at 2 kV. Elemental composition and elemental mapping were performed by Energy Dispersive X-ray analysis (EDX) using the same SEM instrument coupled to a Bruker-X Flash-4010 EDX detector and operating at 10 kV. Alternatively, in some cases SEM images were acquired on a ZEISS GeminiSEM 300. Samples were prepared by drying a diluted dispersion of the MOFs particles in methanol on a silicon wafer substrate.

Transition Electron Microscopy (TEM): A JEOL 2100Plus operated at 200 kV was used to acquire TEM images. A diluted dispersion of the MOFs particles in methanol was placed and let it dry on 200 mesh copper grids coated with Formvar/carbon film.

Nuclear Magnetic Resonance Spectroscopy (NMR): ^{1}H NMR and ^{19}F NMR spectra were recorded on a 400 MHz Bruker Avance III HD spectrometer. Samples were acid digested in a 10% D_2SO_4–DMSO-d_6 mixture, and the NMR spectra of the resulting mixtures were recorded.

X-Ray Diffraction (XRD): X-ray analysis of the powdered samples was performed using a Bruker D8-Advance Diffractometer operated at 50 kV and 1 mA for Cu kα (λ=1.5418 Å). All measurements were collected in 2D mode using a Bruker EIGER-2R 500K Detector (θ =11.9º, γ = 6º), with a step of 11.7º per 300 s in the range 2º-40º (2θ). The detector distance from the samples was 35 cm.

Fourier-transform infrared spectroscopy (FT-IR): Spectra of the samples were recorded using an Jasco FT/IR-4100 spectrometer with a scanner velocity of 2.2 kHz and a resolution of 4 cm^{-1} in the range 400-4000 cm^{-1}. The MOFs particles were deposited on a silicon wafer substrate to obtain the transmittance infrared.

N_2 physisorption analysis: Textural properties of the MOFs particles were evaluated using N_2 adsorption measurements at 77, which were performed in a home-built fully automated manometric equipment from the Advanced Materials Laboratory group. Prior to the adsorption measurements, samples were outgassed at 150 ºC for 12 h under ultra-high vacuum conditions. Specific surface area (S_{BET}) was obtained after application of the Barrett–Emmett–Teller (BET) equation to the N_2 adsorption data. Total pore volume was determined from the amount adsorbed at P/P_0 = 0.97. Micropore volume (V_{micro}) was obtained applying the Dubinin-Radushkevich equation (DR) to the N_2 adsorption data, while mesopore volume (V_{meso}) was obtained from the difference $V_{total-Vmicro}$. Pore size distribution analysis was carried out using

the non-local density functional theory (NLDFT) method to the nitrogen adsorption data (carbon, slit pore, equilibrium model).

Thermogravimetric analysis (TGA): A Mettler Toledo TGA/SDTA instrument was used to perform the thermal profile and the differential scanning calorimetry curves (DSC) of the powdered samples, using a heating profile from 30 to 800 °C (heating rate 10 ºC/min) under an air flow of 100 mL/min.

S2. Synthesis of NU-1000 particles (NU) and post-synthetic fluorination (NU@F)

NU-1000 synthesis: Using as a starting point a procedure previously published,[34] we optimized the experimental conditions to decrease the particle size to *ca.* 700 nm. $ZrOCl_2$.8H2O (784 mg, 2.4 mmol) and benzoic acid (16 g, 131 mmol) were mixed in 64 mL of DMF in a 250-mL round bottom flask and dissolved with the aid of sonication (about 10 min) until achieving a clear solution. The round flask was connected with a condenser, placed in a heating mantle and heated at 100 °C for 1 h under gentle stirring (200 rpm). This resulted in the formation of Zr_6 clusters, [Zr6(μ3-O)4(μ3-OH)4]. After cooling down the solution to RT, H4TBAPy (320 mg, 0.48 mmol; suspended in 32 mL DMF) was added to the round flask, followed by the addition of TFA (320 μL, 4.16 mmol). This reaction mixture was heated up to 100 ºC using a Pt100 thermal sensor for a precise temperature control, and kept at this temperature for 1 h under continuous stirring (400 rpm; a key point to obtain a homogeneous particle size). Afterwards, the reaction mixture was cooled down to RT, and the resulting NU-1000 particles were separated by centrifugation (8000 RCF, 5 min), and washed three times with fresh DMF (15 mL each, using sonication for 30 min between washes). After the last washing step, the resulting yellow powder was suspended in 40 mL DMF.

NU-1000 activation: To remove the coordinated modulators from the node, an HCl washing step was performed as follows. 4 mL of 8 M aqueous HCl was added to the previously obtained suspension of NU-1000 particles in 40 mL of DMF. This mixture was heated overnight (18 h) at 100 ºC with gentle stirring to promote the diffusion of HCl between the particles and through the pores, thus achieving complete elimination of the coordinated molecules. After cooling to RT, the particles were isolated by centrifugation (8000 RCF, 5 min) and washed first with DMF (three times, 15 mL each, 30 min sonication between washes) and second with MeOH (three times, 15 mL each, 30 min sonication between washes). Finally, the purified NU-1000 crystals were collected by centrifugation, dried in an oven at 80 ºC for 1 h, and further thermally activated in a vacuum oven at 120 ºC for 18 h (yield: 420 mg activated NU-1000).

Fluorination of NU-1000 (NU@F): The functionalization of the particles with the fluorinated ligand, specifically perfluorodecanoic acid (PFDA) was carried out by following a previously reported procedure, but scaling up all reagents accordingly.[26] Briefly, 400 mg of activated NU-1000 (0.18 mmol) was mixed with 16 mL of 0.1M solution of PFDA (1.6 mmol) in a glass vial and closed with a cap septum. The mixture was heated in an oven at 60 ºC for 24 h. After that, the resulting NU@F particles were isolated by centrifugation (8000 RCF, 5 min), washed two times with hot DMF (10 mL each, 10 min sonication between washes) and washed then two times with MeOH (10 mL each, 10 min sonication between washes). Finally, the purified NU@F particles were collected by centrifugation and dried in an oven at 80 ºC overnight.

S3. Morphological/structural characterization of NU and NU@F

Additional characterization data (not included in the main manuscript) of the as-prepared NU and NU@F particles are presented in the following.

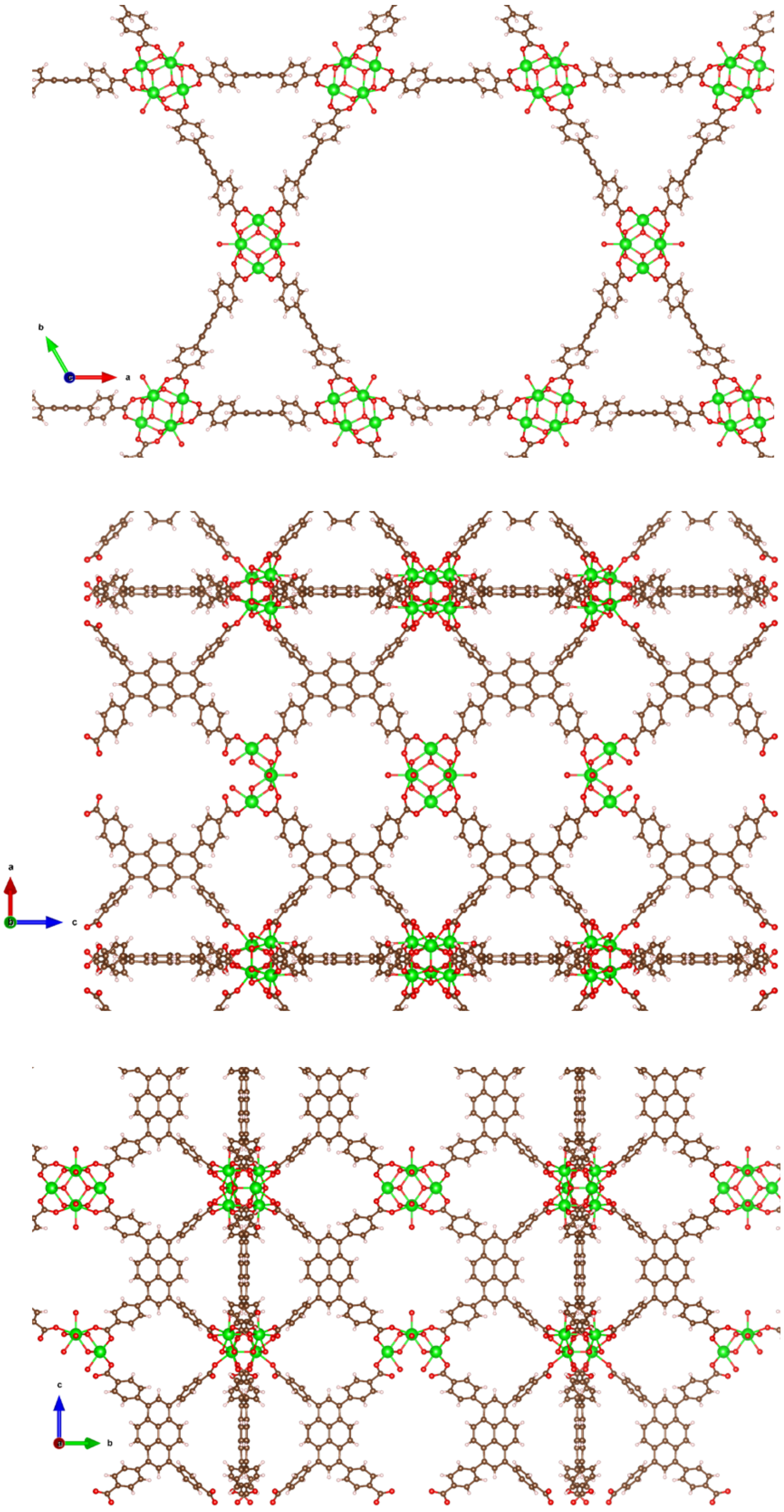

Figure S1. The crystal structure of NU-1000 viewed along different directions. The hexagonal and triangular channels within the framework are clearly visible in the view along the *c* direction. Redrawn from CCDC deposition 7230579.

The morphology (shape and size) and the homogeneity of the as-prepared particles were investigated with SEM and TEM, see Figure S2.

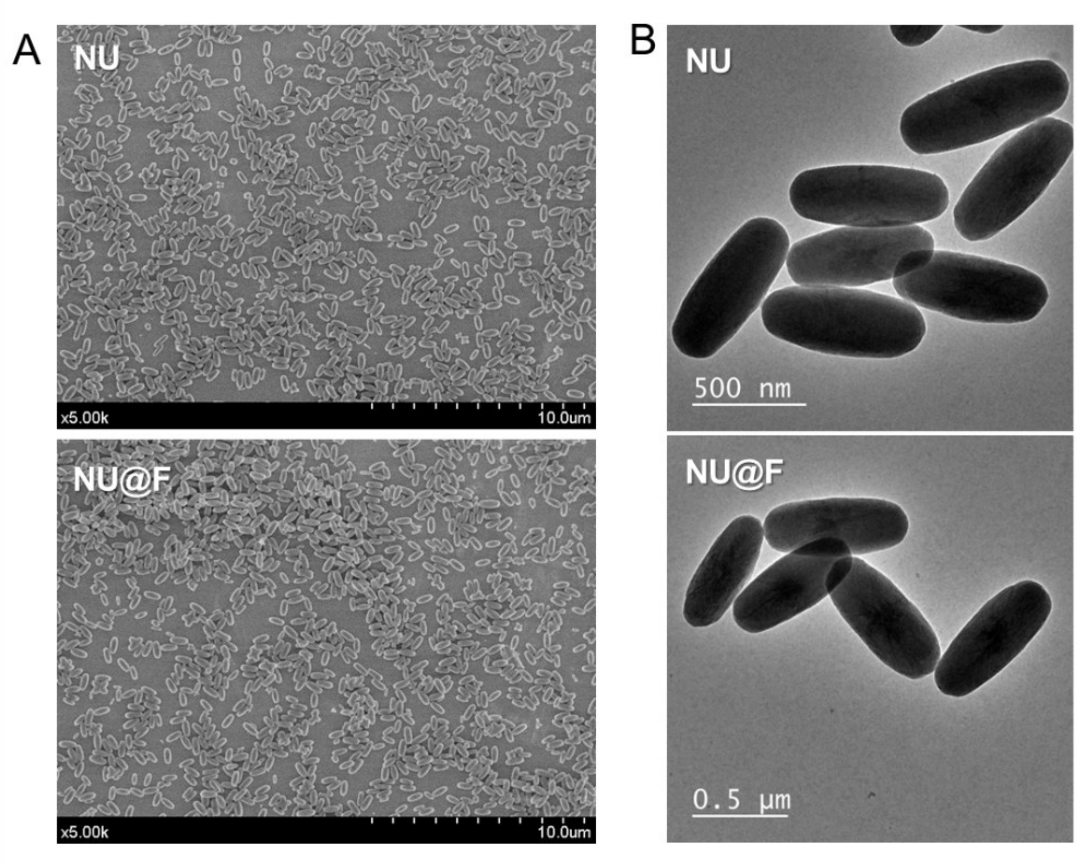

Figure S2. SEM (A) and TEM (B) images with different magnifications to clearly see the homogeneity of the as-prepared NU and NU@F particles.

The coordination between the Zr_6 clusters and the carboxylate groups of the H_4TBAPy ligands was confirmed by FT-IR, as shown in Figure S3. The H_4TBAPy ligands exhibited two characteristic bands corresponding to the C=O stretching (1685 cm^{-1}) and C–O stretching (1370 cm^{-1}) vibrational modes of their carboxylic acid groups. Upon coordination of the $TBAPy^{4-}$ ligand to the metal centers, these bands completely disappeared in the spectrum of the NU particles, and a new band appeared at 1411 cm^{-1}, attributed to the ν(COO−) stretching. A comparison of the FT-IR spectra between NU and NU@F revealed no significant changes in either spectrum, indicating that the structure of the material remained unaltered. It is noteworthy that there was an increase in the band at 1411 cm^{-1}, which can be attributed to the carboxylate groups from PFDA coordinated with the Zr_6 clusters. New bands in the range 1120-1260 cm^{-1}, attributed to the symmetric and asymmetric stretching vibrational modes of the -CF_2- groups of PFDA chains, confirmed the successful incorporation of PFDA into the NU-1000 framework.

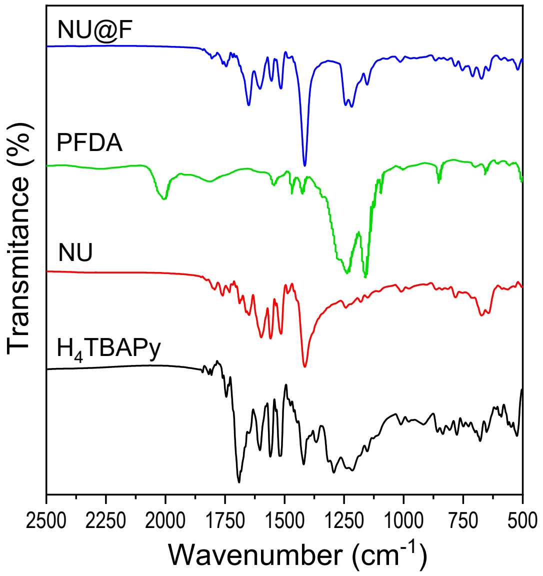

Figure S3. FT-IR spectra of the as-prepared NU and NU@F particles, showing also the spectra of the organic ligands incorporated within the particles.

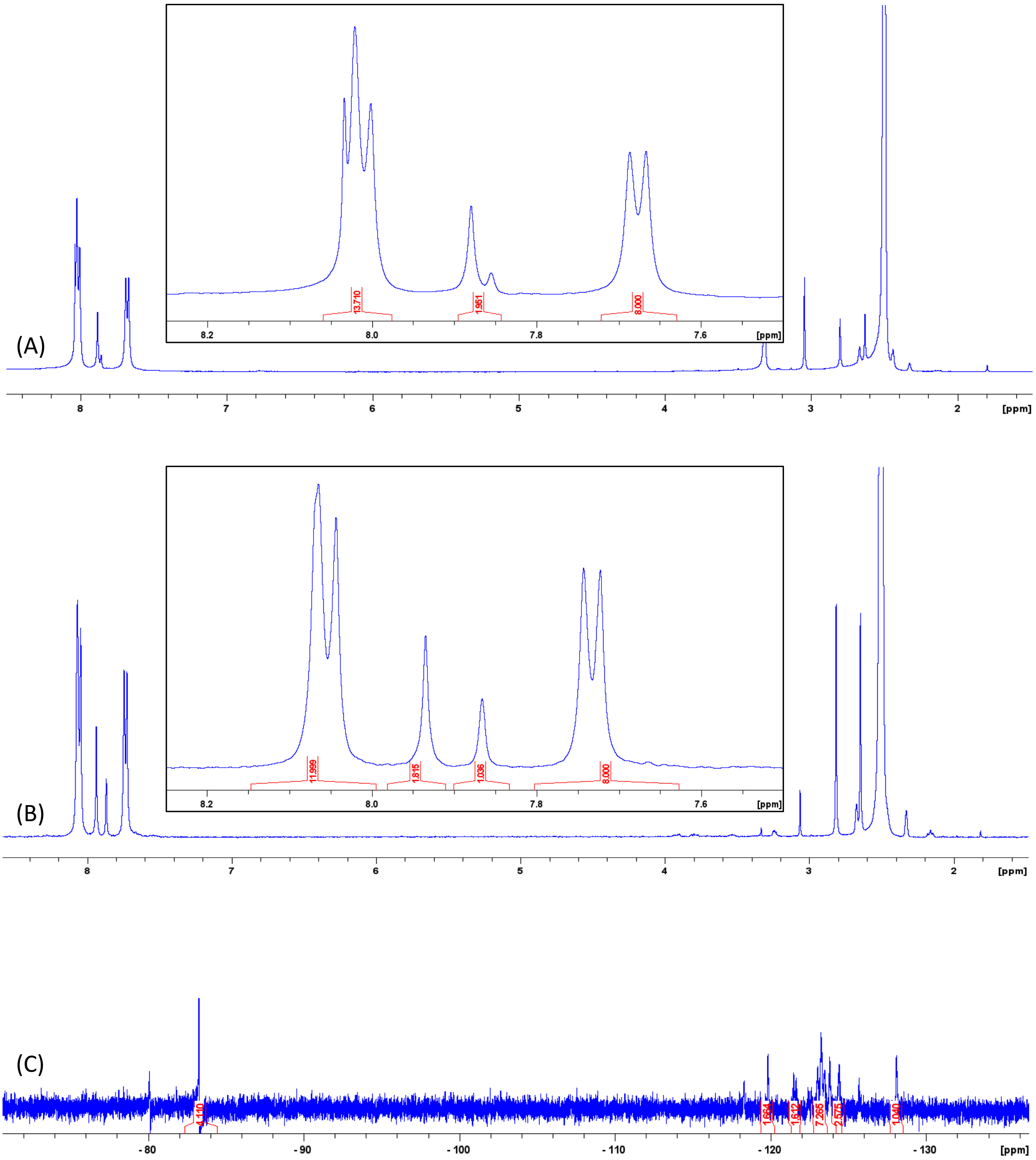

Figure S4. SEM-EDX elemental mapping (recorded at 10 kV for 30 min), where is clearly visible the homogeneous distribution of all the elements in the NU and NU@F particles, as well as the corresponding EDX spectra (recorded at 10 kV for 5 min). (A) ^{1}H NMR spectrum of NU sample. (B), (C) ^{1}H NMR and ^{19}F NMR spectra of NU@F sample. Samples were decomposed in 10% D_2SO_4/DMSO-d_6 mixture.

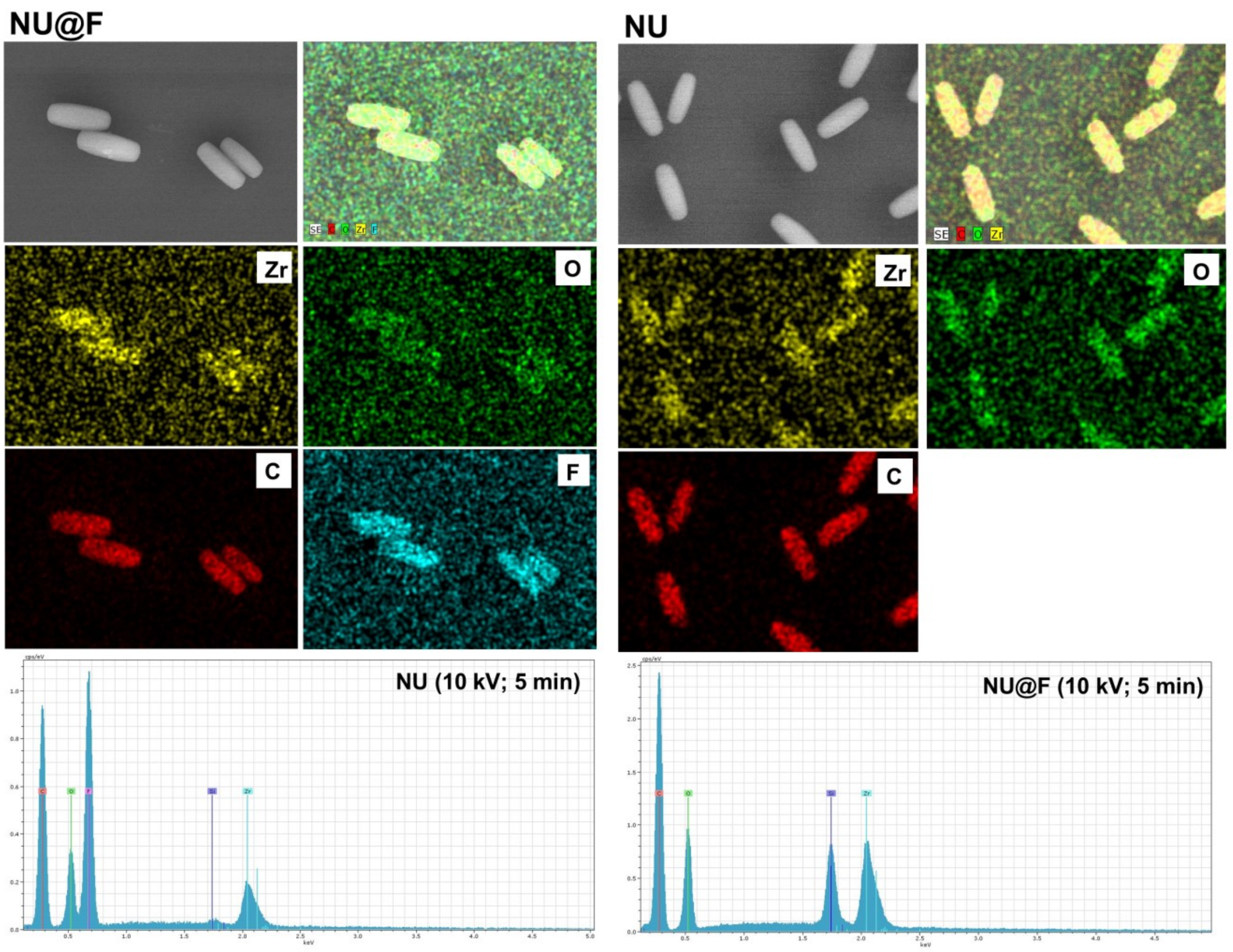

Figure S5. SEM-EDX elemental mapping (recorded at 10 kV for 30 min), where is clearly visible the homogeneous distribution of all the elements in the NU and NU@F particles, as well as the corresponding EDX spectra (recorded at 10 kV for 5 min).

Table S1. Miller indices (hkl), reflection angles (2θ) and interplanar spacing d (d-space), calculated using Bragg's law, of the first ten reflection peaks of the diffractograms shown in Figure 1C,D.

	Simulated		NU		NU@F	
**(hkl)**	**2θ (º)**	**d (Å)**	**2θ (º)**	**d (Å)**	**2θ (º)**	**d (Å)**
100	2.60	34.01	2.568	34.375	2.583	34.177
110	4.50	19.63	4.519	19.537	4.463	19.781
200	5.19	17.00	5.158	17.120	5.149	17.151
001	5.33	16.57	5.222	16.908	5.390	16.384
101	5.93	14.89	5.867	15.052	5.977	14.775
210	6.87	12.85	6.940	12.726	6.992	12.632
201	7.44	11.87	7.417	11.909	7.458	11.845
300	7.79	11.34	7.725	11.435	7.714	11.451
211	8.70	10.16	8.687	10.171	8.711	10.143
220	9.00	9.82	9.064	9.749	8.923	9.902
400	10.40	8.50	10.35	8.540	10.297	8.584
221	10.47	8.45	10.462	8.449	10.431	8.474

Table S2. Textural properties of the as-prepared NU and NU@F particles, calculated from the N_2 isotherms shown in Figure 2A,B.

**Sample**	**S_{BET} ($m^2 \cdot g^{-1}$)**	**V_{total} ($cm^3 \cdot g^{-1}$)**	**V_{micro} ($cm^3 \cdot g^{-1}$)**	**V_{meso} ($cm^3 \cdot g^{-1}$)**	**Mesopore width (nm)**
NU	2235	1.58	0.90	0.68	3.1
NU@F	1097	0.75	0.42	0.33	2.9

S_{BET}: Surface area calculated by BET equation; V_{micro}: micropore volume estimated using DR equation; V_{meso}: mesopore volume calculated from V_{total}-V_{micro}. Mesopore width estimated using NLDFT method.

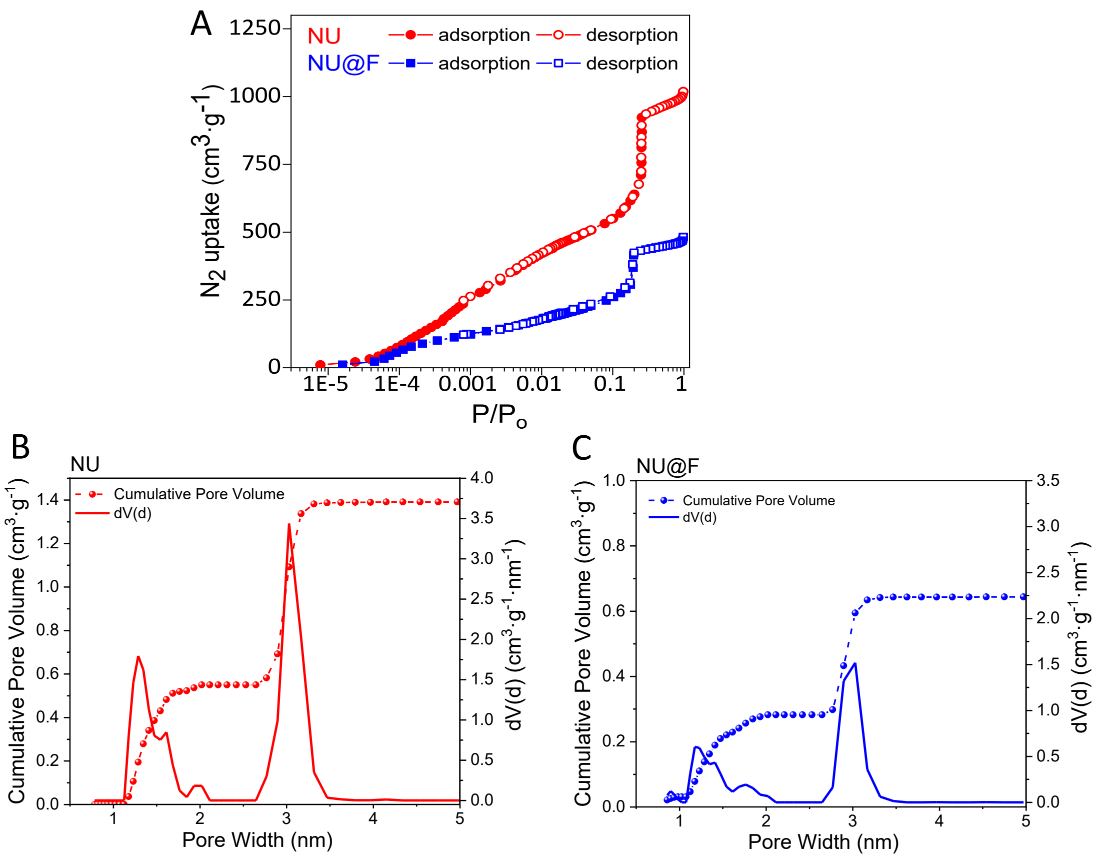

Figure S6. (A) N_2 isotherms at 77 K of NU and NU@F on a semi-logarithmic scale to clearly see the low-pressure behavior of the particles before and after functionalization. NLDFT pore size distribution analyses of the (B) NU and (C) NU@F particles, showing the cumulative pore volume and the micropores/mesopores distribution.

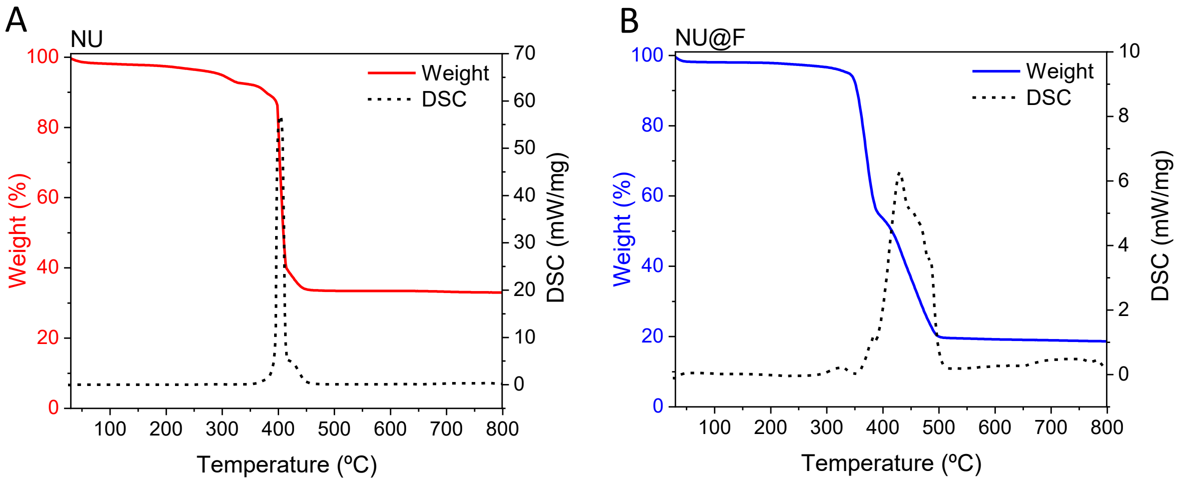

Figure S7. TGA and DSC curves of the (A) NU and (B) NU@F particles.

S4. Quantification of toxins by HPLC-MS

High performance liquid chromatography (HPLC) analyses were carried out on a Bruker Elute UHPLC system consisting of a binary pump, column oven, and an autosampler. The reverse phase chromatography was performed with an analytical X Select HSST3 (100Å, 2.5 µm, 2.1 mm x 150 mm, 100 µm particle × 2,1 mm, 2,5 µm; from Waters), which was kept at 40°C, while the autosampler was maintained at 8°C. The optimized method used a binary gradient mobile phase with water and 0.1 % formic acid as mobile phase A, and acetonitrile with 0.1% formic acid as mobile phase B. The gradient program is shown in Table S3. The flow rate was 0.3 mL/min and the injection volume was 5 µL. For the detection, the HPLC system was coupled to a mass spectrometer detector (MS), AmaZon SL Dual Fuel Ion Trap MS equipped with Compass HyStar software. The mass spectrometer conditions are shown in Table S4. All samples were run in triplicate.

Table S3. HPLC mobile phase gradient program conditions for the analysis of uremic toxins.

	Time (min)	A (%)	B (%)
0.3	0	100	0
0.3	0.5	100	0
0.3	7	0	100
0.3	11	0	100

Table S4. ESI-MS spectrometer conditions.

ESI Source	negative
Capillary	4.50 kV
Nebulizer	1.50 bar
Dry Gas	10 L/min
Dry Temperature	335 ºC
Target Mass	250 m/z
Mass Scan	200-2200 m/z

To determine the concentration of both toxins (pCS and IS) after performing the adsorption experiments, the collected solutions (i.e., supernatants in the experiments under static conditions, or perfusate after passing through the MOF-cartridge in the experiments in flow; corresponding to the non-adsorbed fraction) were analysed by HPLC-MS using the described method and concentrations were calculated by interpolation of the areas to previously constructed analytical calibration curves. Calibration curves were constructed by plotting peak area against the corresponding concentration (mM) of each calibration standard (Figures S8-S10). The removal efficiency was calculated using the following Equation 1:

$$Removal\ (\%) = \frac{C_i - C_f}{C_i} x\ 100 \qquad \text{Equation 1}$$

where C_i = the initial concentration and C_f = the final concentration.

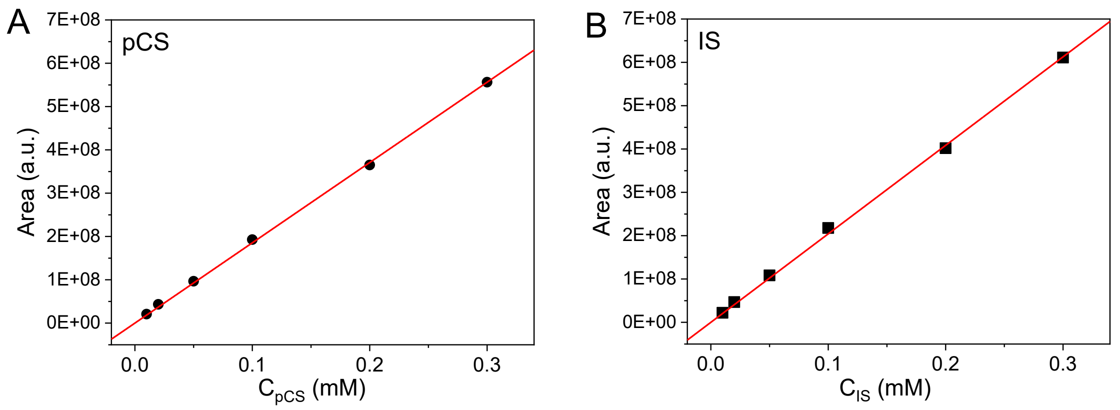

Equation	y = a + b*x
Plot	Area (a.u.) vs C (mM)
Intercept (a)	0
Slope (b)	1.854E9 ± 1.44E7
R-Square	0.9997

Equation	y = a + b*x
Plot	Area (a.u.) vs C (mM)
Intercept (a)	0
Slope (b)	2.042E9 ± 2.06E7
R-Square	0.9995

Figure S8. Calibration curves of (A) pCS and (B) IS as obtained from HPLC-MS measurements of standard solutions. Area as a function of concentration is plotted for each toxin, and calibration equations are obtained by fitting a linear regression line to the data.

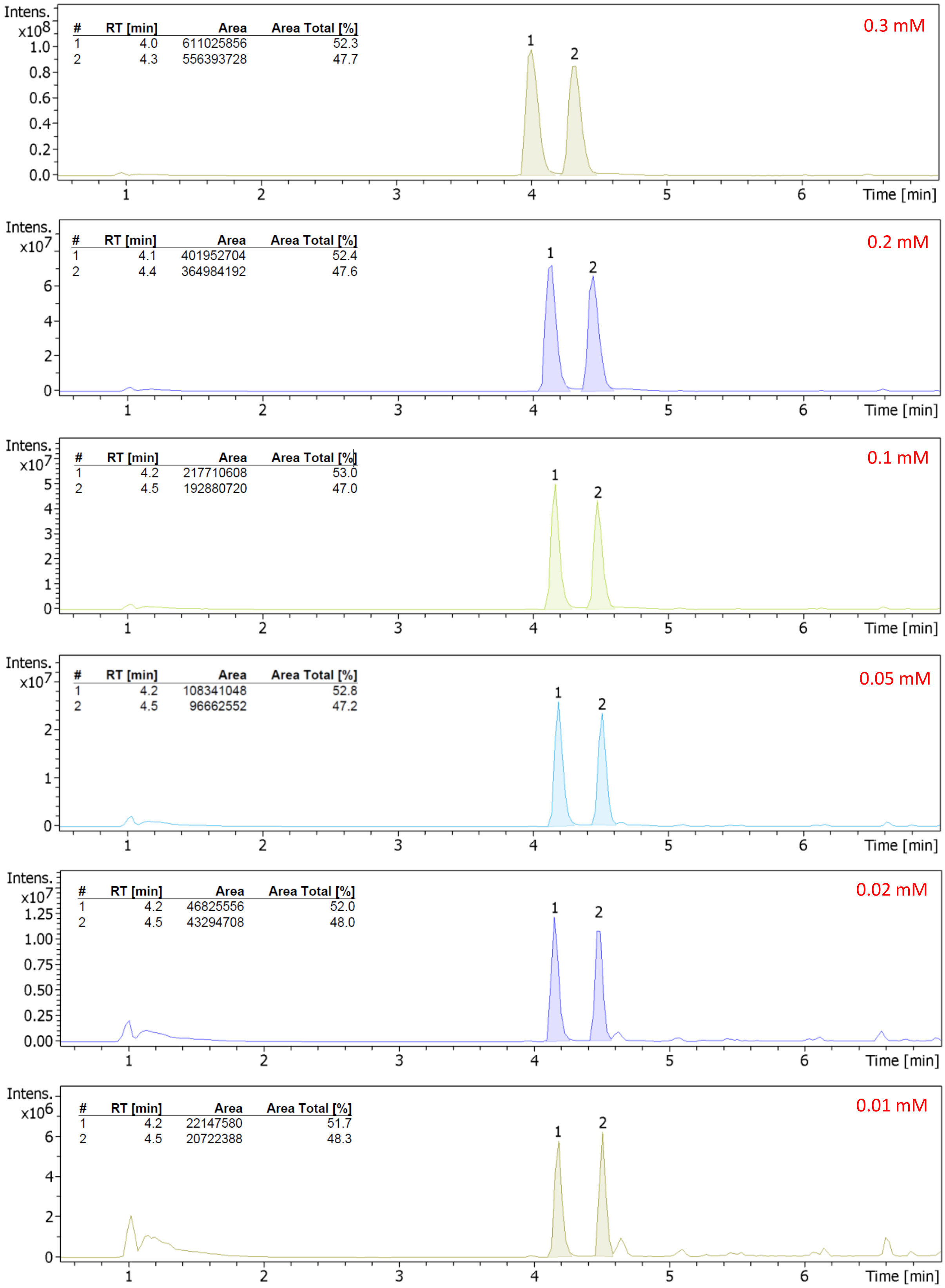

Figure S9. HPLC chromatograms obtained with the calibration standards (pCS and IS, ranging from 0.01 to 0.3 mM; in PBS 10 mM at pH=7.4) and used for preparing the calibration curves shown in Figure S7. Measurements were performed in triplicate and the average areas were plotted. Peak 1 corresponds to IS and peak 2 to pCS (as confirmed by MS spectrum; Figure S9).

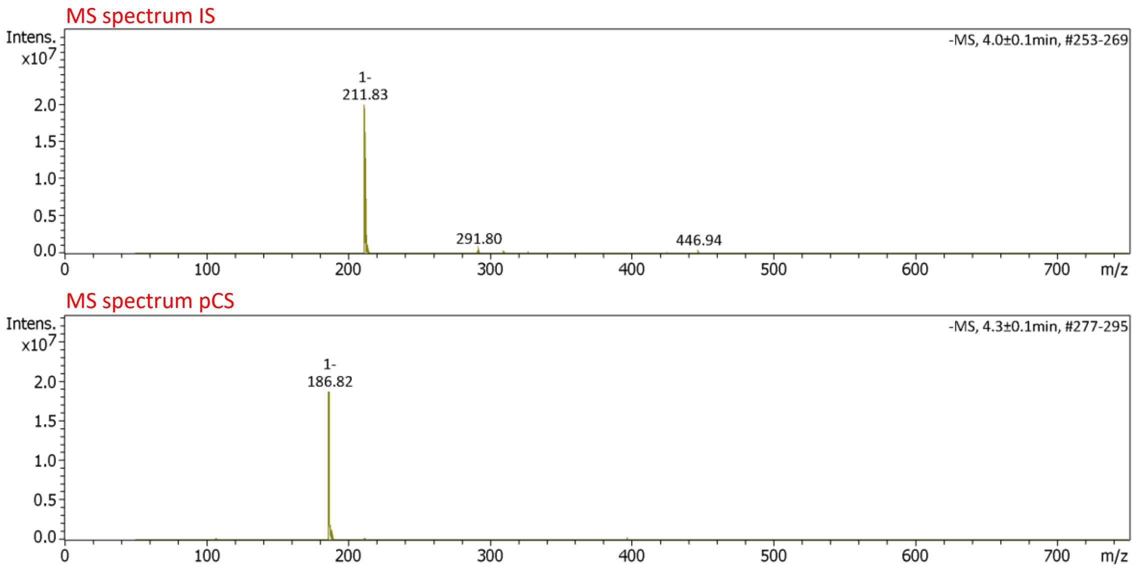

Figure S10. The MS spectrum of each toxin in negative ionization mode, which enable unequivocal identification of the target toxins.

S5. Adsorption experiments under static conditions

Adsorption studies in the absence of HSA. Adsorption experiments were performed by exposing 10 mg of NU or NU@F particles with 6.5 mL of a PBS solution containing both toxins at 0.3 mM each in 12-mL glass vials, and the mixture was incubated under static conditions at RT (~22ºC) for 24 h (unless otherwise specified) to reach saturation uptake. The concentration of pCS and IS was set at 0.3 mM, because this is the maximum possible concentration found in end-stage CKD patients, and the toxins solution in PBS was freshly prepared before use. Note that the NU and NU@F particles remained suspended quite homogeneously in the toxins solution during incubation (Figure S11). After incubation, the MOF particles were separated by centrifugation (8000 RCF, 5 min), and the amount of toxins adsorbed on the MOFs was quantified indirectly, by measuring the toxins remaining in the supernatants by HPLC-MS (as described above). The removal efficiency was calculated by Equation 1 (Table S5).

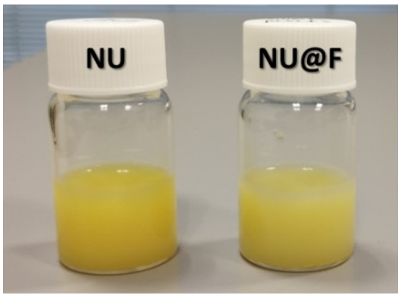

Figure S11. Photograph of the NU and NU@F particles incubated with the solution of toxins (0.3 mM each, PBS 10 mM pH=7.4).

To investigate the uptake of uremic toxins as a function of time, kinetic studies were performed by following the same procedure but varying the incubation time (up to 48 h). The removal efficiency as a function of time was measured by HPLC-MS and calculated by Equation 1 (Table S5).

Reusability. The reusability studies were performed by collecting the MOF particles by centrifugation after first use, washing the particles twice with methanol and twice with PBS, and resuspending the final pellet (regenerated particles) in a fresh solution of toxins for the second adsorption assay. The MOFs were reused three times, and the removal after each reuse were determined by HPLC-MS as previously described (Table S5).

Table S5. Performance of NU and NU@F particles for the removal of free uremic toxins under different conditions.

MOF sample	Toxin / C (mM), V (mL) / medium	MOF amount (mg)	MOF amount corrected* (mg)	Incubation time (h) / T (º C)	Removal** (%)	Uptake capacity (mmol/mg)	Uptake corrected* (mmol/mg)
NU	pCS / 0.3, 6.5 / PBS	10	−	24 / 22	78.1	152.3	−
NU	IS / 0.3, 6.5 / PBS	10	−	24 / 22	50.7	98.8	−
NU@F	pCS / 0.3, 6.5 / PBS	10	8.6	24 / 22	69.0	134.5	156.4
NU@F	IS / 0.3, 6.5 / PBS	10	8.6	24 / 22	46.1	89.7	104.3
NU	pCS / 0.3, 6.5 / PBS	10	−	1 / 22	15.2	29.6	−
NU	IS / 0.3, 6.5 / PBS	10	−	1 / 22	11.3	22.0	−
NU@F	pCS / 0.3, 6.5 / PBS	10	8.6	1 / 22	6.2	12.1	14.1
NU@F	IS / 0.3, 6.5 / PBS	10	8.6	1 / 22	4.3	8.4	9.7
NU	pCS / 0.3, 6.5 / PBS	10	−	3 / 22	48.2	94.0	−
NU	IS / 0.3, 6.5 / PBS	10	−	3 / 22	36.2	70.6	−
NU@F	pCS / 0.3, 6.5 / PBS	10	8.6	3 / 22	17.4	33.9	39.4
NU@F	IS / 0.3, 6.5 / PBS	10	8.6	3 / 22	15.2	29.6	34.5
NU	pCS / 0.3, 6.5 / PBS	10	−	6 / 22	72.2	140.8	−
NU	IS / 0.3, 6.5 / PBS	10	−	6 / 22	49.2	95.9	−
NU@F	pCS / 0.3, 6.5 / PBS	10	8.6	6 / 22	37.2	72.5	84.3
NU@F	IS / 0.3, 6.5 / PBS	10	8.6	6 / 22	25.9	50.5	58.7
NU	pCS / 0.3, 6.5 / PBS	10	−	12 / 22	78.0	152.1	−
NU	IS / 0.3, 6.5 / PBS	10	−	12 / 22	51.4	100.2	−
NU@F	pCS / 0.3, 6.5 / PBS	10	8.6	12 / 22	53.2	103.7	120.6
NU@F	IS / 0.3, 6.5 / PBS	10	8.6	12 / 22	37.5	73.1	85.0
NU	pCS / 0.3, 6.5 / PBS	10	−	48 / 22	79.2	154.4	−
NU	IS / 0.3, 6.5 / PBS	10	−	48 / 22	50.2	97.9	−
NU@F	pCS / 0.3, 6.5 / PBS	10	8.6	48 / 22	67.1	130.8	152.1
NU@F	IS / 0.3, 6.5 / PBS	10	8.6	48 / 22	46.2	89.7	104.7
NU	pCS / 0.1, 3.0 / water	10	−	24 / 22	89.0	178.0	−
NU_R1	pCS / 0.3, 6.5 / PBS	10	−	24 / 22	78.6	153.3	−
NU_R2	pCS / 0.3, 6.5 / PBS	10	−	24 / 22	72.0	140.4	−
NU_R3	pCS / 0.3, 6.5 / PBS	10	−	24 / 22	49.3	96.1	−
NU_R1	IS / 0.3, 6.5 / PBS	10	−	24 / 22	50.7	98.8	−
NU_R2	IS / 0.3, 6.5 / PBS	10	−	24 / 22	43.2	84.2	−
NU_R3	IS / 0.3, 6.5 / PBS	10	−	24 / 22	23.3	45.4	−
NU@F_R1	pCS / 0.3, 6.5 / PBS	10	8.6	24 / 22	67.3	131.2	152.5
NU@F_R2	pCS / 0.3, 6.5 / PBS	10	8.6	24 / 22	63.1	123.0	143.1
NU@F_R3	pCS / 0.3, 6.5 / PBS	10	8.6	24 / 22	64.8	126.4	146.9
NU@F_R1	IS / 0.3, 6.5 / PBS	10	8.6	24 / 22	43.4	84.6	98.4
NU@F_R2	IS / 0.3, 6.5 / PBS	10	8.6	24 / 22	44.2	86.2	100.2
NU@F_R3	IS / 0.3, 6.5 / PBS	10	8.6	24 / 22	42.5	82.9	96.4

*Considering only the fraction of NU-1000 framework (assuming 14.3 wt% of perfluoroalkyl-ligands as determined by TGA)
**The relative standard deviations (RSD) calculated from three independent experiments were < 5% in all cases.

Adsorption studies in the presence of HSA. Uremic toxins (0.3 mM pCS and 0.3 mM IS) were pre-incubated with 0.6 mM HSA for 24 h at RT in PBS solution, allowing the formation of toxin-HSA complexes. Next, this toxin-HSA mixture was subjected to adsorption experiments by either NU or NU@F particles following the same procedure described above. Removal values and uptake capacities under these experimental conditions are given in Table S6. To quantify properly the total amount of toxins (free + protein-bound) by HPLC-MS analyses in the initial and final solutions after adsorption, a sample pretreatment step was carried before injection in the HPLC system, in order to denature and precipite the HSA proteins. For that, samples were deproteinized by the addition of 3 parts of cold methanol to 1 part of sample followed by centrifugation (15000 RCF, 10 min, 4 ºC), and then were analysed by HPLC-MS using the same optimized method described in section S4.

Table S6. Performance of NU and NU@F particles for the removal of total uremic toxins (free + HSA-bound) under static conditions.

MOF sample	Toxin / C (mM), V (mL)	MOF amount (mg)	MOF amount corrected* (mg)	Time (h) / T (º C)	Removal** (%)	Uptake capacity (mmol/mg)	Uptake corrected* (mmol/mg)
NU	pCS-HSA / 0.3, 6.5	10	−	24 / 22	47.5	92.6	−
NU	IS-HSA / 0.3, 6.5	10	−	24 / 22	31.3	61.0	−
NU@F	pCS-HSA / 0.3, 6.5	10	8.6	24 / 22	92.3	179.9	209.3
NU@F	IS-HSA / 0.3, 6.5	10	8.6	24 / 22	88.2	172.0	200.0

*Considering only the fraction of NU-1000 framework (assuming 14.3 wt% of perfluoroalkyl-ligands as determined by TGA)
**The relative standard deviations (RSD) calculated from three independent experiments were < 7% in all cases.

S6. Structural changes in NU and NU@F particles after toxins removal

Post-characterization of the used NU and NU@F were carried out to evaluate the potential structural changes upon adsorption of the uremic toxins in PBS in both the presence and absence of HSA, since proteins may also affect to the MOF's structure. To this end, the MOF particles were collected by centrifugation after toxins adsorption and analysed by SEM, FTIR, and XRD. Besides Figures 3D-F and Figure 4C (included in the main manuscript), some additional post-characterization data are presented below.

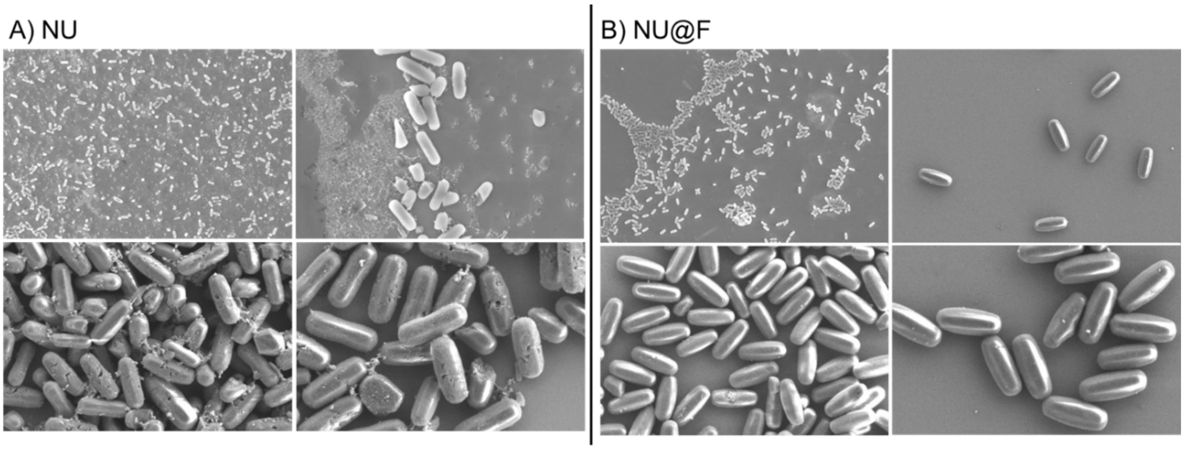

Figure S12. SEM images of the (A) NU and (B) NU@F particles after 24 h incubation with a PBS solution containing uremic toxins (pCS and IS; 0.3 mM each). Images at different magnifications and using two detectors (InLens or SE2) are shown to clearly see the generation of defects on the pristine NU-1000 (i.e., NU particles) after the adsorption process.

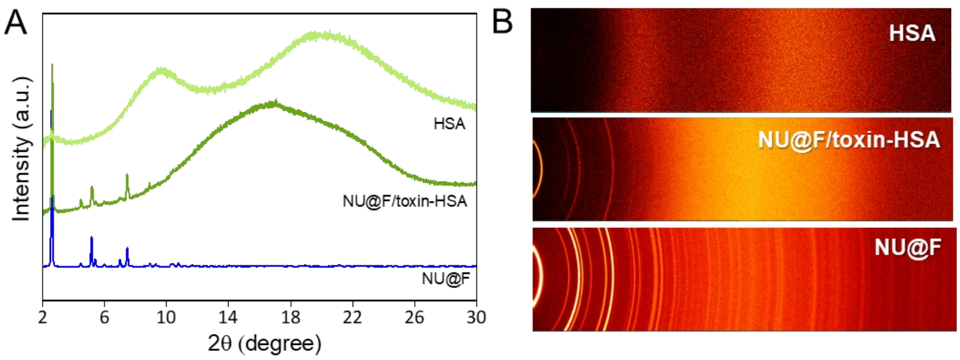

Figure S13. PXRD spectra and 2DXRD images of NU and NU@F particles before and after adsorption experiment of toxin-HSA complexes. The PXRD and 2DXRD of lyophilized HSA is also shown.

The crystalline index (CI) was used to quantitatively evaluate the changes in the crystallinity of the MOF particles after toxins adsorption in different conditions (Table S7). The CI is defined as the ratio of the crystalline domain ($I_{crystalline}$) and the total domain (crystalline and amorphous phases) of a material ($I_{crystalline} + I_{amorphous}$), and is calculated by using the Equation 2.

$$CI\ (\%) = \frac{I_{crystalline}}{I_{crystalline} + I_{amorphous}} \times 100 \qquad \text{Equation 2}$$

Table S7. CI values of NU and NU@F particles, before and after adsorption experiments

Adsorption experiment conditions	CI (%)			
	NU		NU@F	
	before	after	before	after
Free toxins (0.3 mM each) in PBS; 24 h incubation	85 ± 3	45 ± 8	75 ± 2	72 ± 5
Toxin-HSA (0.3 mM each toxin; 0.6 mM HSA) in PBS; 24 h incubation	85 ± 3	38 ± 5	75 ± 2	56 ± 6

* CI values are shown as mean ± SD from three independent XRD measurements

Note that the slight reduction in crystallinity for the as-synthetized NU@F compared to pristine NU can be attributed to the incorporation of the long-fluorinated organic chains of PFDA. Likewise, the decrease in crystallinity in NU@F particles after incubation in the presence of HSA is due to the incorporation of the low-crystalline/amorphous HSA protein into the system, which decreases the overall crystallinity. Note that prior to these XRD studies, the particles were collected just by centrifugation and not washed to remove the potential proteins adsorbed on the MOF surface. The HSA used had a CI of 21%, reflecting its inherently amorphous nature.

S7. Adsorption experiments under flow using the dialysis setup

Dialysis setup. A prototype of MOF-cartridge was built by using a reservoir with luer lock connectors to contain the MOFs particles (Figure S14A). At both ends of the reservoir, a 0.45 µm pore size nylon hydrophilic membrane/filter (#7402-001; Cytiva Europe GmbH) was placed to prevent any potential leakage of the MOF particles. For each experiment, 30 mg of NU@F were suspended in 200 µL of methanol and sonicated for proper dispersion. Then, 200 µL of PBS was added, and the suspension was transferred into the reservoir. The reservoir contents were manually perfused with 5 mL of PBS to remove methanol. The NU@F cartridge was integrated in a closed-circuit perfusion system (Figure S14B) connected to a peristaltic pump for the continuous perfusion of the toxins' solution through the cartridge at 24 mL/min flux for 24 h, allowing in this way the cumulative adsorption of toxins on the MOFs within the cartridge.

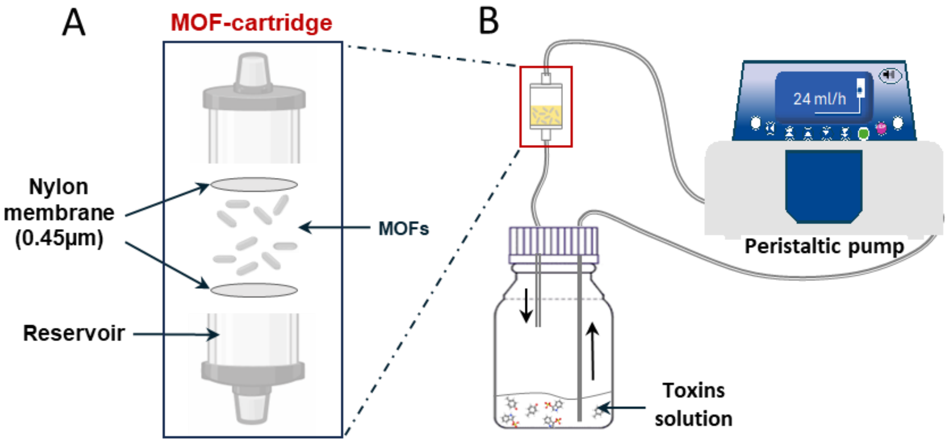

Figure S14. Schemes of the (A) MOF-cartridge prototype, and (B) closed-circuit perfusion system.

Toxins removal procedure. Adsorption experiments under flow conditions were performed within the built dialysis setup, and served to evaluate the performance of the NU@F-cartridge for the removal of uremic toxins. For that, 20 mL of either solution of free toxins (0.3 mM pCS, 0.3 mM IS, in PBS) or HSA-toxins mixture (0.3 mM pCS, 0.3 mM IS, 0.6 mM HSA in PBS; mixture preincubated 24 h at RT) was pumped at 24 mL/min flux through the NU@F-cartridge (containing 30 mg NU@F) for 24 h. After this time, the concentration of toxins remaining in the perfused solutions were quantified by HPLC-MS as described above and compared with the initial concentration to determine the removal efficiencies (Table S8). Three independent experiments were conducted for each condition.

Reusability of the MOF-cartridge. Reusability studies were conducted to evaluate the removal efficiency and stability of the MOF particles in flow after multiple uses. For this purpose, after each use, the NU@F particles were washed by perfusing methanol for 2 h, followed by PBS for 1 h at a flow rate of 24 mL/h. Subsequently, a fresh solution of HSA-toxins was perfused. Three reuses were carried out. The perfused solutions collected after each use were quantitatively analyzed by HPLC-MS (Table S8). Three independent experiments were conducted for each condition.

Table S8. Performance of NU@F-cartridge particles for the removal of free uremic toxins (pCS, IS) or total uremic toxins (free + HSA-bound) under flow conditions and several reuses (R1, R2 and R3 correspond to first second, and third use of the sample NU@F-cartridge).

MOF sample	Toxin / C (mM), V (mL)	MOF amount (mg)	MOF amount corrected* (mg)	Time (h) / T (º C)	Removal** (%)	Uptake capacity (mmol/mg)	Uptake corrected* (mmol/mg)
NU@F-cartridge	pCS / 0.3, 20	30	25.7	24 / 22	60.6	121.2	141.5
NU@F-cartridge	IS / 0.3, 20	30	25.7	24 / 22	43.1	86.2	100.6
NU@F-cartridge_R1	pCS-HSA / 0.3, 20	30	25.7	24 / 22	83.7	167.4	195.4
NU@F-cartridge_R2	pCS-HSA / 0.3, 20	30	25.7	24 / 22	79.5	159.0	185.6
NU@F-cartridge_R2	pCS-HSA / 0.3, 20	30	25.7	24 / 22	75.1	150.2	175.3
NU@F-cartridge_R1	IS-HSA / 0.3, 20	30	25.7	24 / 22	73.5	147.0	171.6
NU@F-cartridge_R2	IS-HSA / 0.3, 20	30	25.7	24 / 22	68.7	137.4	160.4
NU@F-cartridge_R3	IS-HSA / 0.3, 20	30	25.7	24 / 22	64.4	128.8	150.3

*Considering only the fraction of NU-1000 framework (assuming 14.3 wt% of perfluoroalkyl-ligands as determined by TGA)
**The relative standard deviations (RSD) calculated from three independent experiments were < 8% in all cases.

S8. Studies with human samples from patients

All procedures were performed in accordance with the ethical standards of the institutional research committee of IMIBIC (Maimonides Biomedical Research Institute of Cordoba), and complying with the standards established by the latest revision of the Declaration of Helsinki and in the Council of Europe Convention (1996), concerning Human Rights and Biomedicine. The study protocol was approved by the local institutional ethics committee (Comité de Ética de la Investigación de Córdoba; ref. 5479, protocol code PI-0130-2022). All patients provided written informed consent to participate in the study.

Ultrafiltrate sample from CKD patients. A pooled human real sample, consisted of ultrafiltrate sample from CKD patients undergoing hemodialysis, was used to evaluate the actual potential of our proposed NU@F-dialysis treatment. Specifically, hemodiafiltration with endogenous reinfusion (HFR) was employed to obtain such endogenous ultrafiltrate (or plasma water; UF). HFR is a renal replacement therapy that uses a double-chamber dialyzer plus an adsorptive cartridge.[51]. The blood passes through a super-high-flux membrane with an area of 0.7 m², characterized by a high cut-off (>55,000 Da), which allows ultrafiltration by means of a pressure gradient. This process generates an UF that contains significant concentrations of medium-high weight molecules, such as protein-bound uremic toxins, indoxyl sulfate (IS 212,200 Da) and p-cresyl sulfate (pCS 188,200 Da). Ultrafiltrate samples were collected during the initial three minutes of the first weekly dialysis session, prior to passage through the adsorbent cartridge, to ensure a high concentration of uremic solutes.

Toxins removal procedure. The pooled human sample was subjected to the dialysis treatment using the NU@F-cartridge and following the experimental conditions indicated in section S7. The sample was analyzed by HPLC-MS before and after such NU@F-dialysis treatment to quantify the amount of toxins and determine the removal efficiency for each toxin (Figure S15, Table S9). After that, the sample was fortified with additional amounts of both toxins (pCS and IS, 0.3 mM each) to simulate the worst conditions, that is plasma samples in end-stage CKD patients before undergoing HFR, and this fortified sample was also subjected to the NU@F-dialysis treatment, and analyzed by HPLC-MS (Table S9).

Table S9. Removal efficiency of the target uremic toxins (pCS, IS) in a human real sample () by using the developed NU@F-dialysis system.

Sample	Toxin / C_i (mM)	C_i (mM)*	C_f (mM)*	Removal*** (%)
Real sample (ultrafiltrate)	pCS; unknown	0.0230 ± 0.0031	< LOD**	100
Real sample (ultrafiltrate)	IS; unknown	0.0120 ± 0.0028	< LOD**	100
Fortified real sample	pCS; initial + 0.3	0.326 ± 0.018	0.081 ± 0.016	75.1
Fortified real sample	IS; initial + 0.3	0.318 ± 0.011	0.111 ± 0.004	65.0

*C_i and C_f are the concentrations of toxin before and after treatment determined by HPLC.
**LOD = limit of detection.
***RSD values from three independent experiments were < 5% in all cases.

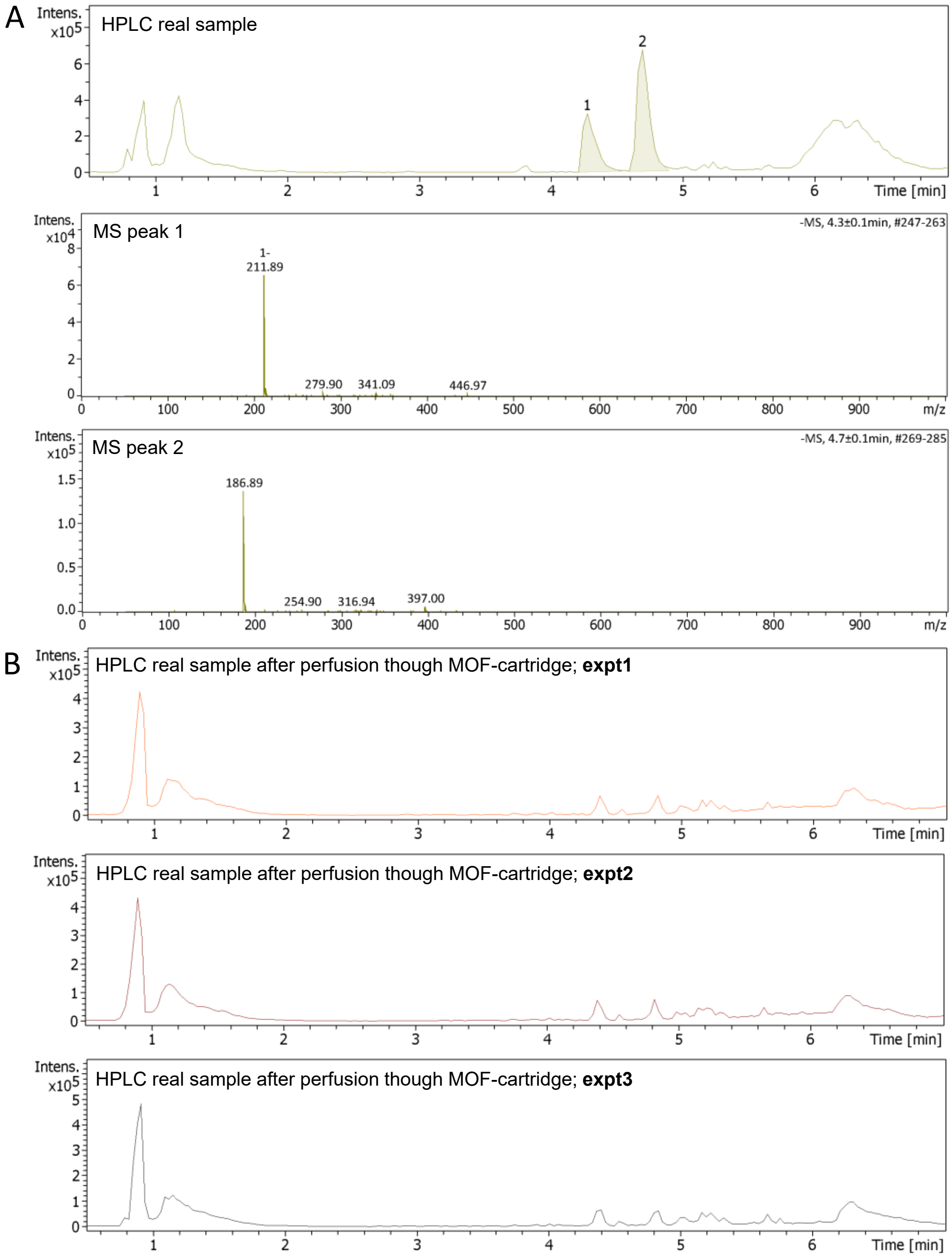

Figure S15. (A) HPLC chromatogram of real sample and the corresponding MS spectra of the two target toxins detected (1=IS; 2=pCS). (B) HPLC chromatograms of the same sample after being subjected to the NU@F-dialysis system for the removal of the toxins; the chromatograms from three independent experiments are shown.

Comparison of ESI modes. The ESI negative ionization is beneficial in the case of real complex samples such as human plasma, as the vast majority of the components in biological samples can only be positively ionized, giving rise to HPLC-MS chromatograms with many peaks, as shown in Figure S16. In contrast, negative ionization is much more selective, leading to chromatograms with fewer possible interferences, which facilitates the detection of the target uremic toxins (pCS and IS), and thus leads to lower detection and quantification limits.

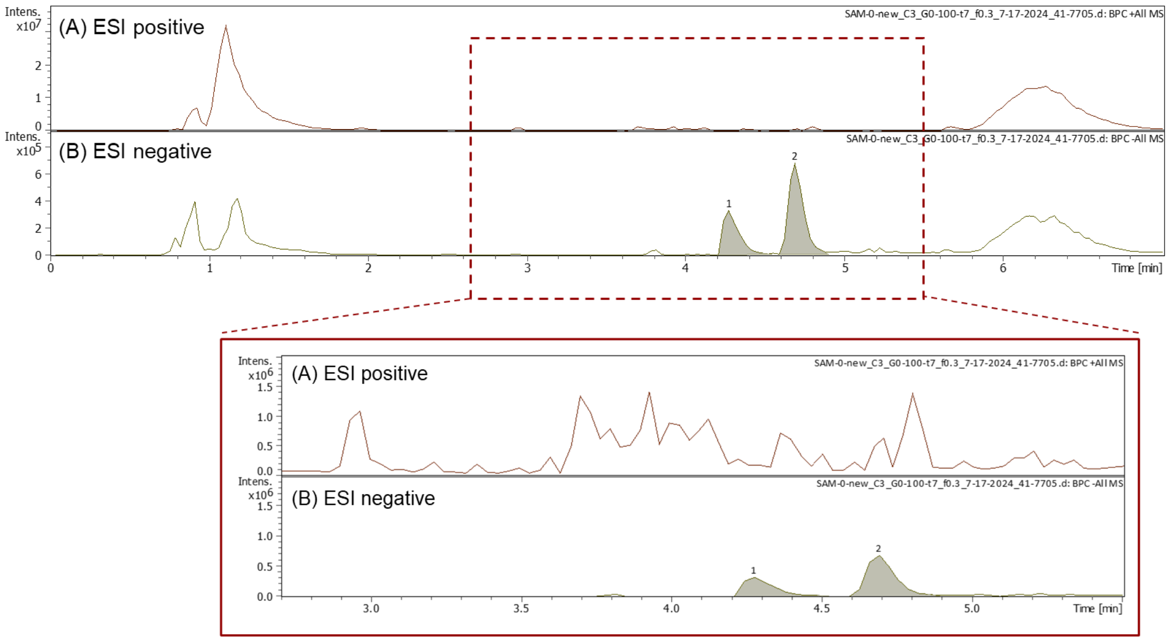

Figure S16. HPLC-MS chromatograms of the real sample obtained in (A) ESI positive ionization and (B) negative ionization. Inset shows a magnification of the chromatograms within the retention time range close to the target toxins (1=IS; 2=pCS) and presented at the same intensity scale (y-axis) to clearly see the presence of many peaks from the multiple components in plasma when recorded at the ESI positive ionization.

S9. Protein quantification and cell viability assays

Protein quantification. The total concentration of proteins in solution was determined by the Bio-Rad protein assay, a colorimetric method based on the Bradford dye-binding technique. The procedure was conducted in accordance with the manufacturer´s instructions. Briefly, dilutions of a protein standard (Merck Group) and sample solutions were mixed with Bradford reagent (#5000006; Bio-Rad Laboratories Inc.), and the absorbance was measured at wavelength of 595 nm using a PowerWave XS microplate spectrophotometer (BioTek Instruments). The protein concentration of the samples was calculated using the standard curve obtained for dilutions of the protein standard.

Cell viability. Human umbilical vein endothelial cells (HUVECs) were seeded in completed medium in 96-well plates at a density of 15,000 cells/well for 24 h. After that, the medium was removed, and fresh medium containing increasing doses of the perfused samples (i.e., samples collected after perfusion through the NU@F-cartridge for 24 h) was added to cells and incubated for 24 h. The viability of these treated cells was determined by WST-1 assay. For that, after treatment the culture medium was replaced with 100 µL of phenol red-free medium, following by the addition of 10 µL of pure WST-1 (Roche) per well. After 2h incubation at 37ºC and 5% CO_2, absorbance was measured at wavelength 450 nm for samples and 600 nm for background using a microplate reader. Cell viability (Figure S17) was calculated by subtracting the background absorbance values from the resulting values of the samples, and data are presented as fold change from the negative control (cells not treated with the perfused sample).

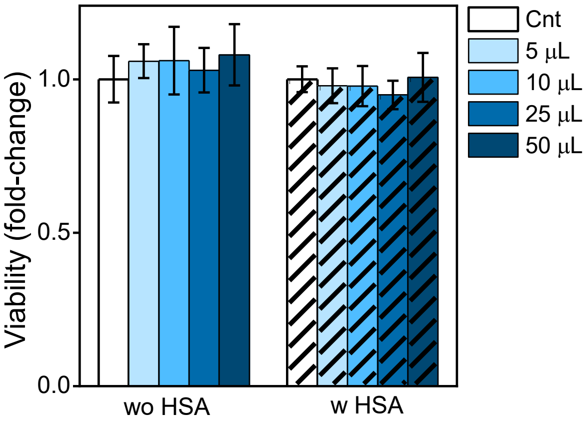

Figure S17. Cell viability of HUVEC cells under 24 h exposure to increasing amounts of the samples collected after perfusion through the NU@F-cartridge for 24 h in two types of samples: solution of free toxins (without HSA; wo HSA) and solution of HSA-toxins complexes (with HSA; w HSA).
